# Liposome-based high-throughput and point-of-care assays toward the quick, simple, and sensitive detection of neutralizing antibodies against SARS-CoV-2 in patient sera

**DOI:** 10.1007/s00216-023-04548-3

**Published:** 2023-02-09

**Authors:** Simon Streif, Patrick Neckermann, Clemens Spitzenberg, Katharina Weiss, Kilian Hoecherl, Kacper Kulikowski, Sonja Hahner, Christina Noelting, Sebastian Einhauser, David Peterhoff, Claudia Asam, Ralf Wagner, Antje J. Baeumner

**Affiliations:** 1 Institute of Analytical Chemistry, Chemo- and Biosensors, University of Regensburg, Universitaetsstr. 31, 93053 Regensburg, Germany; 2 Institute of Medical Microbiology & Hygiene, Molecular Microbiology (Virology), University of Regensburg, Universitaetsstr. 31, 93053 Regensburg, Germany; 3 Mikrogen GmbH, Floriansbogen 2-4, 82061, Neuried, Germany; 4 Institute of Clinical Microbiology and Hygiene, University Hospital Regensburg, Regensburg, Germany

**Keywords:** Liposomes, Neutralizing antibodies, Point-of-care diagnostics, High-throughput screening, SARS-CoV-2

## Abstract

The emergence of severe acute respiratory syndrome-related coronavirus 2 (SARS-CoV-2) in 2019 caused an increased interest in neutralizing antibody tests to determine the immune status of the population. Standard live-virus-based neutralization assays such as plaque-reduction assays or pseudovirus neutralization tests cannot be adapted to the point-of-care (POC). Accordingly, tests quantifying competitive binding inhibition of the angiotensin-converting enzyme 2 (ACE2) receptor to the receptor-binding domain (RBD) of SARS-CoV-2 by neutralizing antibodies have been developed. Here, we present a new platform using sulforhodamine B encapsulating liposomes decorated with RBD as foundation for the development of both a fluorescent, highly feasible high-throughput (HTS) and a POC-ready neutralizing antibody assay. RBD-conjugated liposomes are incubated with serum and subsequently immobilized in an ACE2-coated plate or mixed with biotinylated ACE2 and used in test strip with streptavidin test line, respectively. Polyclonal neutralizing human antibodies were shown to cause complete binding inhibition, while S309 and CR3022 human monoclonal antibodies only caused partial inhibition, proving the functionality of the assay. Both formats, the HTS and POC assay, were then tested using 20 sera containing varying titers of neutralizing antibodies, and a control panel of sera including prepandemic sera and reconvalescent sera from respiratory infections other than SARS-CoV-2. Both assays correlated well with a standard pseudovirus neutralization test (*r* = 0.847 for HTS and *r* = 0.614 for POC format). Furthermore, excellent correlation (*r* = 0.868) between HTS and POC formats was observed. The flexibility afforded by liposomes as signaling agents using different dyes and sizes can hence be utilized in the future for a broad range of multianalyte neutralizing antibody diagnostics.

**Graphical abstract:**

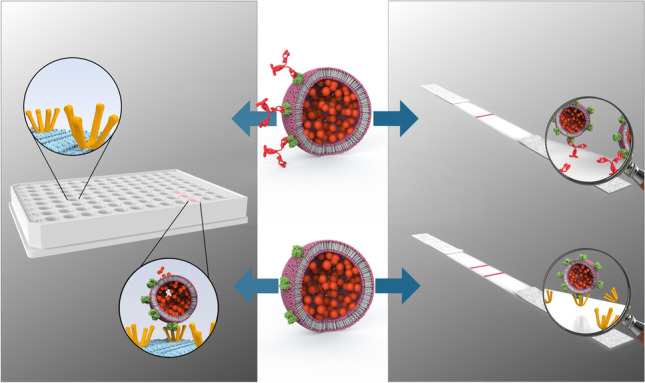

**Supplementary Information:**

The online version contains supplementary material available at 10.1007/s00216-023-04548-3.

## Introduction

COVID-19, a disease caused by the infection with severe acute respiratory syndrome-related coronavirus 2 (SARS-CoV-2), had affected more than 600 million and killed >6 million people worldwide in just 2.5 years. Recovery from an infection coincides with the production of neutralizing antibodies (nAbs) as part of the humoral immune response helping to contain an active infection, reducing the risk for reinfection and contributing toward prevention of severe disease [1, 2]. Similar responses can be induced by the various approved vaccines, in particular spike (S) protein encoding mRNA vaccines (BNT162b2 — BioNTech/Pfizer, mRNA-1273 — Moderna), adenovirus vectored vaccines (ChAdOx1 nCoV-19 — AstraZeneca, Ad26.COV2.S — Johnson&Johnson), protein vaccines (NVX-CoV2373 — NovaVax), and inactivated vaccines (CoronaVac — Sinovac Life Sciences) [3–6]. Compared to commonly used antibody binding assays, neutralizing antibodies represent a superior correlate for protection from infection or severe disease [7]. The gold standard for nAb titer determination is the 50% plaque-reduction neutralization test (PRNT_50_), requiring up to 3 days and a biosafety level 3 (BSL-3) laboratory due to the use of live virus [8]. Alternative cell-based assay formats include pseudovirus neutralization tests (pVNT), only requiring BSL-2 facilities due to the use of lentivirus or vesicular stomatitis virus pseudotyped with SARS-CoV-2 spike protein [9, 10]. These assays quantify the total nAb titer, including all nAbs directed against various domains of the spike (S) protein of SARS-CoV-2, which is responsible for docking of the virus to the host cells and cell entry via membrane fusion.

The S protein consists of two subunits, S1 and S2. The former surface-exposed subunit contains the receptor-binding domain (RBD), which binds specifically to the human angiotensin-converting enzyme 2 (ACE2) receptor [11]. Subsequent cleavage of S1 and S2 by host cell proteases (especially TMPRSS2) activates the S2 subunit, facilitating fusion of viral and host membranes, causing the release of the viral genome into the host cell cytoplasm [12]. The predominant subunit eliciting virus neutralization was found to be the receptor-binding domain [13, 14], thus making the RBD a natural choice for assay development, reinforced even further by higher yields in production compared to recombinant trimeric spike protein. This knowledge enabled the development of surrogate virus neutralization tests (sVNT) based on the competitive binding of nAbs and ACE2 to RBD. These competitive binding assays include both ELISAs for high-throughput analysis in a laboratory setting and paper-based lateral flow assays for point-of-care testing. Different competitive ELISAs relying on horseradish peroxidase (HRP)-conjugated RBD or ACE2 for detection are commercially available, such as the cPass™ SARS-CoV-2 Neutralization Antibody Detection Kit from GenScript®, the SARS-CoV-2 sVNT Kit from ProteoGenix, and the iFlash-2019-nCoV Neutralization Antibody Test from YHLO. Other sensing strategies have been investigated in pursuit of shorter assay times, increased throughput, and improved sensitivity. A flow-based chemiluminescence microarray immunoassay could detect nAbs in blood samples in just 7 min [15], while a four-channel SPR sensor enabled simultaneous detection of anti-S1 antibodies as well as free and neutralized virus particles [16]. The use of a Tri-part split-NanoLuc® facilitated a homogeneous neutralization test [17] and the use of Luminex beads holds the potential for multiplexing [18]. For detection of neutralizing antibody titers at the point-of-care, multiple versions of sVNT lateral flow assays have been proposed, using ACE2 or RBD test lines and different markers conjugated to RBD or ACE2. A photometric read-out is commonly used, enabled with RBD-conjugated gold nanoparticles [19, 20] or green-gold nanoshells [21], ACE2-biotin with streptavidin-conjugated HRP using 3,3′,5,5′-tetramethylbenzidine (TMB) as substrate [22], or cellulose nanobeads conjugated to monoclonal non-neutralizing anti-RBD antibodies [23]. Conjugation of RBD to EuNPs even enabled a fluorescent read-out [24]. For improved sensitivity, a second test line can be added to capture neutralized particles, the signal being directly proportional to the nAb titer, while the intensity of the normal test line is proportional to the inverse of the nAb titer [19, 24].

**Fig. 1:**
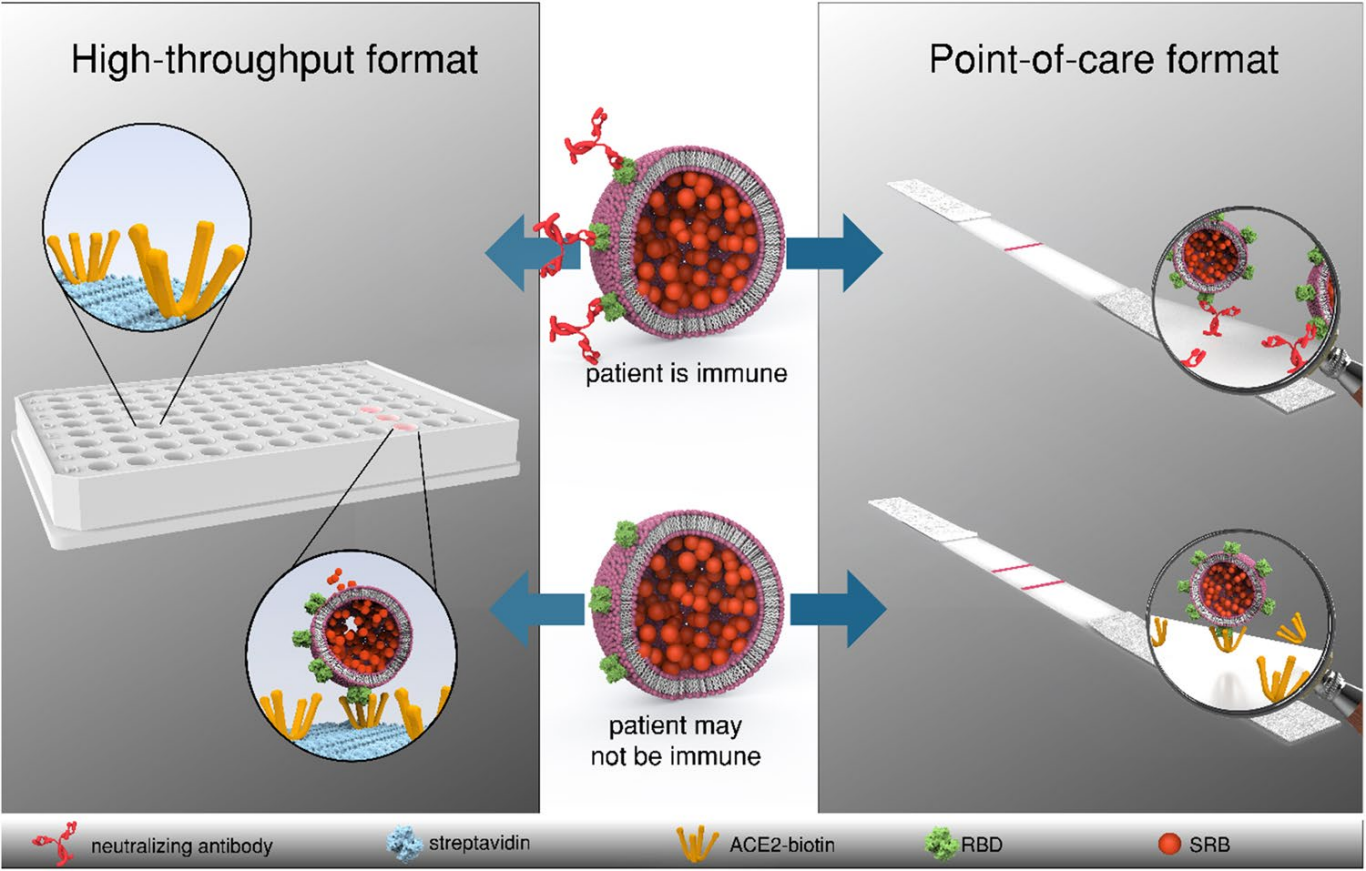
Schematic of the developed high-throughput (i) and point-of care (ii) formats for the detection of neutralizing antibodies directed against the receptor binding domain of SARS-CoV-2. RBD-conjugated liposomes are incubated with patient serum and subsequently (i) immobilized in an ACE2-coated microplate or (ii) mixed with ACE2-biotin and added to a nitrocellulose membrane with streptavidin test line. In presence of neutralizing antibodies, no liposome-binding takes place, and no signals are observed in either format. In the absence of neutralizing antibodies liposomes are (i) bound in the microplate, enabling detection of the released sulforhodamine B via fluorescence after washing and lysis or (ii) bound by the test line, enabling a colorimetric detection via camera or naked eye

Here, we investigated the use of sulforhodamine B (SRB) encapsulating liposomes conjugated to RBD as a signaling alternative. In previous work, SRB liposomes were shown to surpass gold nanoparticles in sensitivity in a lateral flow assay [25]. Additionally, the encapsulated SRB enables the use of the liposomes in fluorescence-based microplate assays. This feature allows for the use of one marker in two different assay formats such as ELISA and lateral flow assay, facilitating better comparison of the two while reducing fabrication steps (Fig. 1). The presence of carboxylic acid groups on the liposomal surface facilitates protein modification via simple coupling strategies, such as EDC/NHS chemistry, and the large inner cavities allow for encapsulation of different markers, e.g., *m*-carboxyluminol [26] or redox markers such as ferri/ferro hexacyanide [27], and hence easily support chemiluminescent, electrochemical, or optical multianalyte approaches.

## Experimental section

### Chemicals and consumables

All chemicals were of analytical reagent grade. Cholesterol from sheep wool (C8667, ≥ 99%), *N*-hydroxysulfosuccinimide sodium salt (sulfo-NHS), purity ≥ 98%, fluorescein 5(6)-isothiocyanate (FITC) (≥ 90%, 46950), and Sephadex-G50 were purchased from Sigma Aldrich/Merck (Darmstadt, Germany); 1,2-dipalmitoyl-sn-glycero-3-phosphoethanolamine-*N*-(glutaryl) (sodium salt) (*N*-glutaryl-DPPE) from Coatsome; the remaining phospholipids 1,2-dimyristoyl-sn-glycero-3-phosphoethanolamine (DMPE), 1,2-dipalmitoyl-sn-glycero-3-phosphocholine (DPPC), 1,2-dipalmitoyl-sn-glycero-3-phospho-(1′-rac-glycerol) (sodium salt) (DPPG), and the extruder set from Avanti Polar Lipids (Alabaster, AL, USA). Sulforhodamine B (SRB) (S1307), (1-ethyl-3-(3-dimethylaminopropyl) carbodiimide-hydrochloride) (EDC) (PG82079), and neutralizing SARS-CoV-2 spike protein (RBD) Polyclonal Antibodies (PA5-114451) were purchased from Thermo Fisher Scientific (Germany); *n*-Octyl-β-D-glucopyranoside (OG) (≥ 98%, CN23), 2-(N-morpholino)-ethane sulphonic acid (MES) (≥ 99%, 4259), and N-2-Hydroxyethylpiperazine-N′-2-ethane sulphonic acid (HEPES) (≥ 99.5%, HN78) from Carl Roth (Karlsruhe, Germany). Custom lateral flow test strips (streptavidin test line, <anti-FITC> control line, CN150 membrane) (LFP-915) and standard capacity streptavidin microplates (604500) were purchased from Microcoat Biotechnologie GmbH (Bernried, Germany). For additional information on common reagents and buffer compositions, see SI.

### Recombinant proteins

Monoclonal human anti-RBD IgG1 antibodies (S309) (BYT-ORB746635) were purchased from Biozol (Eching, Germany). All recombinant proteins were expressed in different scales using the commercial Expi-Fectamine™ system (Thermo Fisher Inc.) according to manufacturer’s instructions. Monoclonal human anti-RBD IgG1 antibody (CR3022) was purified from cell culture supernatants using HiTrap Protein A HP column (Cytiva) as described [28]. RBD B1.1.7 with C-terminal Avi-His6 tag was purified using a HisTrap excel column (Cytiva) with a linear gradient of 10 column volumes from phosphate-buffered saline to phosphate buffered saline with 500 mM Imidazole essentially as described earlier [28]. The external domain of ACE2 (amino acids 18-740) with C-terminal Avi-His6 tag was purified using a HisTrap excel column (Cytiva) with a linear gradient of 10 column volumes from phosphate-buffered saline to phosphate buffered saline with 500 mM imidazole. Afterwards, the buffer was exchanged to 20 mM Bistris with 10 mM NaCl, pH 6.8, and the protein was further polished using HiTrap DEAE FF column, with a linear gradient from 10 to 1000 mM NaCl in 20 mM BisTris, pH 6.8. All proteins were buffer exchanged to phosphate-buffered saline and stored at 4 °C.

ACE2 was site-specifically biotinylated at the Avi-tag using BirA biotin-protein ligase standard reaction kit (Avidity) according to the manufacturer protocol.

Proteins were quality controlled with SDS-PAGE. Binding was confirmed by a slightly modified ELISA protocol [20] using biotinylated ACE2 and streptavidin-HRP (1:5000, Roche) instead of antibodies.

### Antisera

Sera were obtained from the seroprevalence cohort TiKoCo-19, provided by Mikrogen GmbH or purchased. In brief, TiKoCo19 samples comprised sera after SARS-CoV-2 acquisition, vaccination, or both. Multiple serological tests were applied to determine seropositivity [29, 30]. The TiKoCo-19 study was approved by the Ethics Committee of the University of Regensburg, Germany (vote 20-1867-101). The study complies with the 1964 Helsinki declaration and its later amendments. All participants provided written informed consent. Additional sera from COVID-19 reconvalescent as well as vaccinated individuals were collected in the Munich area (“Munich Cohort”) after a call for voluntary donation of serum samples for serological analysis related to SARS-CoV-2. The samples were drawn by the family doctors, according to the legal specifications communicated to us by the Ethics commission of the Bavarian Medical Board [Ethik-Kommission der Bayerischen Landesärztekammer](R008-067 mat/ch), based on §24 MPG (Medizinproduktegesetz) and European norm http://eur-lex.europa.eu/legal-content/DE/TXT/?uri=OJ:L:2017:117:TOC. Volunteers gave their written consent for testing. The logistic support of Mikrogen GmbH collected the anonymized sera and all necessary information. The use of sera given by human research participants has been performed in accordance with the Declaration of Helsinki. Prepandemic pooled human complement serum (ICSER, seronegative PHS) was purchased from Innovative Research (Novi, Michigan, USA). Seronegative prepandemic anonymized plasma samples from healthy adult blood donors (blood donations) were purchased from the Bavarian Red Cross. RSV, influenza A, adenovirus, and mycoplasm seropositive samples were purchased from commercial vendors.

### Liposome synthesis

Liposomes were synthesized using the reverse-phase evaporation method as described previously [31]. The encapsulant (4.5 mL) was prepared by dissolution of SRB and NaCl in 20 mM HEPES, pH 7.5, at 60 °C using sonication. Lipids (Table S1) were dissolved in 3 mL chloroform and 0.5 mL methanol and sonicated for 1 min. 2 mL encapsulant were added to the lipid mixture and the solution was sonicated for 4 min at 60 °C. Organic solvents were evaporated at a rotary evaporator (LABOROTA 4001, Heidolph, Germany) at 60 °C by stepwise reduction of pressure (900 mbar for 10 min, 850 mbar for 5 min, 800 mbar for 5 min, 780 mbar for 20 min). The solution was vortexed for 1 min, another 2 mL of encapsulant were added, and the solution was vortexed again for 1 min. The residual organic solvents were evaporated at 60 °C (750 mbar for 20 min, 600 mbar for 5 min, 500 mbar for 5 min, 400 mbar for 20 min). The solution was then extruded using polycarbonate membranes with pore sizes of 1 μm, 0.4 μm, and 0.2 μm. Extrusion was conducted at 65 °C with repeated pushing of the syringes, amounting to 21 repetitions for the 1 μm pore size and 11 repetitions for each of the smaller pore sizes. Excess encapsulant was then removed by size exclusion chromatography using a Sephadex G-50 column, followed by dialysis overnight against HSS buffer with 2 buffer exchanges in a dialysis membrane Spectra/Por© 4 (MWCO: 12-14 kDa).

### Characterization of liposomes

The phospholipid concentration was determined using an inductively coupled plasma optical emission spectrometer (ICP-OES) (SpectroBlue TI/EOP) from SPECTRO Analytical Instruments GmbH (Kleve, Germany). Phosphorous standard dilutions between 0 μM and 100 μM in 0.5 M HNO_3_ were used for calibration of the device. Phosphorous was detected at 177.495 nm. Before each measurement, 0 μM and 100 μM phosphorus dilutions were used to re-calibrate the device. Liposome stock solutions were diluted 1:100 in 0.5 M HNO_3_ and their phosphorous content determined. Total lipid concentrations were calculated using the phospholipid concentration and the lipid composition used during synthesis.

Size and ζ-potential were measured via dynamic light scattering (DLS) using a Malvern Zetasizer Nano-ZS. Liposome stock solutions were diluted 1:100 in HSS buffer in a polymethyl methacrylate (PMMA) semi-micro cuvette (Brand, Germany) for size and a disposable folded capillary cell (Malvern Panalytical, Germany) for ζ-potential measurements. The measurement temperature was set to 25 °C, the refractive index was 1.34, the material absorbance was zero, and the dispersant viscosity 1.1185 mPa s. For ζ-potential, a dielectric constant of 78.5 was used and an equilibration time of 60 s applied before each measurement.

### Modification of liposomes

RBD was conjugated to carboxylated liposomes via EDC/NHS chemistry. First, liposomes were incubated for 1 h at room temperature (RT) and 300 rpm with EDC and sulfo-NHS (1:100:180 ratio of carboxy-groups: EDC:sulfo-NHS). The desired amount of protein was added, and the solution incubated for another 1.5 h at RT and 300 rpm. The reaction was quenched by addition of 10 mM l-lysine-hydrochloride. Finally, the solution was dialyzed against HSS buffer overnight with one buffer exchange in a Spectra-Por® Float-A-Lyzer® G2 (1 mL, MWCO: 1000 kDa).

For FITC conjugation, liposomes were dialyzed against a carbonate buffer (100 mM NaHCO_3_, 250 mM NaCl, pH 9) overnight. FITC dissolved in DMSO (1 mg/mL) was added to the liposomes (1:50 ratio of amine groups to FITC) and incubated overnight at RT and 300 rpm. The solution was dialyzed against HSS buffer overnight with three buffer exchanges in a dialysis membrane Spectra/Por© 4 (MWCO: 12–14 kDa). Total lipid concentrations were determined using ICP-OES and the conjugated liposomes were stored at 4 °C.

### HTS neutralization test

If not stated otherwise, all microtiter plate-based assays were run according to the following protocol. A standard capacity streptavidin microplate was coated with ACE2-biotin (1 μg/mL in PBS, 100 μL) for 1 h at RT and 300 rpm. Meanwhile, liposomes (0.5 μM total lipids) were incubated with serum dilutions (4 v%, followed by 2-fold series dilution) in HSS buffer for 1 h at 30 °C and 300 rpm in a ThermoMixer C (Eppendorf SE, Germany). The plate was washed two times with PBS-T (150 μL) and three times with HSS (150 μL) before addition of the liposome-serum mixture (100 μL). After 2 h incubation at RT and 300 rpm, the plate was washed three times with HSS buffer (150 μL). Captured liposomes were lysed by 10 min incubation with 30 mM OG (100 μL). The fluorescence intensities were measured three consecutive times with a BioTek SYNERGY neo2 fluorescence reader (𝜆_𝐸𝑥_ = 560 𝑛𝑚 and 𝜆_𝐸𝑚_ = 585 𝑛𝑚, bandwidth 10, gain 150). Binding inhibition values were calculated as $binding\ inhibition\ \left(\%\right)=\left(1-\frac{fluor.\kern0.5em \mathit{\operatorname{int}}.}{fluor.\kern0.5em \mathit{\operatorname{int}}.\kern0.5em neg.\kern0.5em control}\right)\bullet 100$. Errors were calculated using Gaussian error propagation. IC50 values were determined with GraphPad Prism 9 (GraphPad Software, San Diego, CA, USA) using the “log(inhibitor) vs. normalized response with variable slope” algorithm.

### POC neutralization test

If not stated otherwise, all lateral flow assays were run according to the following protocol. Liposomes were pre-incubated with serum (5 μL) in HSS buffer (47 μL) for 15 min at RT and 300 rpm. ACE2-biotin (3 equivalents per RBD) were added to the mixture (resulting in 50 μL total volume, 10 μM RBD-conjugated liposomes, 12.9 μM FITC-conjugated liposomes, and 10 v% serum). The solution was pipetted into a clear microplate and test strips were dipped into the solutions. After 5 min, the strips were washed using 25 μL HSS buffer. Pictures were taken 20 min after washing buffer addition using a Canon EOS 550D camera with a Canon EFS 18–55 mm lens from 15 cm distance with consistent lighting and the following settings: ISO 100, aperture 3.5, exposure time 1/30 s, focal length 18 mm, and white balance daylight (5200K). The raw files were analyzed using ImageJ. The color channels were split, and the background of the green channel was subtracted for background smoothing (50 pixels) before black/white inversion. The brightness was adjusted to make the lines visible for the naked eye. The intensities of the background, test, and control line were measured threefold using a rectangle of 80 × 45 pixels. The average intensities were calculated and the background signal subtracted. Binding inhibition values were calculated as $binding\ inhibition\ \left(\%\right)=\left(1-\frac{test\ line\ intensity\ }{control\ line\ intensity}\right)\bullet 100$.

### *recom*Line SARS-CoV-2 IgG assay

The commercial *recom*Line SARS-CoV-2 IgG assay (Mikrogen GmbH, Neuried, Germany) was conducted as given in the instructions. Briefly, a nitrocellulose membrane test strip loaded with RBD was incubated with diluted serum or plasma for 1 h and washed. Horseradish peroxidase-conjugated anti-human IgG antibodies were added and incubated for 45 min. The coloring solution containing TMB was added after washing and incubated for 8 min before the signals were analyzed.

### Pseudovirus neutralization test

The pseudovirus-based neutralization assay (pVNT) using the Vesicular Stomatitis Virus (VSV-Δ G*FLuc) pseudotyped with wt-SARS-CoV-2-Spike-ΔER was performed as described earlier [32]. In brief, a fixed inoculum of 25,000 ffu was neutralized with a 2-fold serum dilution series starting from 1 in 20 for 1 h. Luciferase activity was determined 20 h post infection of HEK293T-ACE2-cells using BrightGlo (Promega Corp, Madison, WI, USA).

## Results and discussion

Liposomes were developed to bear the unique property of offering a highly sensitive fluorescent and a rapid, simple colorimetric read-out. This was accomplished using the encapsulation of SRB as previously demonstrated [25, 31, 33]. Key assay development points described here were the investigation of RBD, ACE2 and liposome concentration, ACE2 immobilization, and the optimization of SRB liposomes with respect to signaling strength. For the proof-of-principle assay, binding and neutralizing antibodies as well as seronegative and neutralizing sera were used. Finally, the screening of a serum panel consisting of 20 neutralizing sera previously analyzed by a standard pseudovirus neutralization test was performed.

Recombinant RBD B1.1.7 and biotinylated ACE2 was produced as described above and quality was controlled by SDS-PAGE and ELISA (Fig. S1). SDS-PAGE revealed purity of the proteins (Fig. S1A and B) and ELISA confirmed rigid binding of biotinylated ACE2 to RBD B1.1.7 with a dissociation constant of 5.4 nM ± 3 (Fig. S1C).

### High-throughput format (HTS)

Universal liposomes were synthesized and adapted toward the specific detection of neutralizing SARS-CoV-2 antibodies (nAb) in a competitive assay format. Initially, the optimum RBD coverage of the liposome surface regarding immobilization efficiency was investigated. Between 0.1 mol% and 0.5 mol% RBD were coupled to the liposomes via EDC/NHS chemistry and the modified liposomes were immobilized in an ACE2-coated plate. Best results were achieved with 0.2 mol% RBD, while 0.5 mol% RBD resulted in decreased signal intensities (Fig. S2). This could be either due to crosslinking of excess RBD to RBD coupled to the liposomal surface, as the excess EDC and NHS is not removed before addition of RBD, or the RBD is packed too densely. Both would result in steric hindrance, diminishing the liposomes binding potential to ACE2. In case of the ACE2 concentration used for coating of the microplate, it was found that higher concentrations lead to more liposome binding (Fig. S3). Finally, for the encapsulant concentration, it was found that 50 mM SRB encapsulant led to significant signal improvements over liposomes with 10 mM SRB and allowed the detection at various liposome concentrations (Fig. S4). In later studies, this concentration was further increased to 150 mM SRB (the maximum possible concentration still leading to colloidally, long-term, and serum stable liposomes) to obtain an even more sensitive test. In initial studies for the detection of nAbs, titration curves of nAbs, binding antibodies, and non-neutralizing sera were measured. Expected results were obtained for the antibodies studied (Fig. S5). Signals obtained for the serum samples on the other hand showed a clear interference of the sera with the assay as lower serum concentrations (<0.1%) increased the overall fluorescent signals, whereas higher concentrations lead to a decrease. It is assumed that serum proteins adsorb to the microplate’s surface, despite the use of BSA and other blocking agents, and interact with the released SRB after liposome lysis, decreasing the hydration-mediated quenching [34]. This leads, in general, to an increase of the overall fluorescence signal. Yet, at higher serum concentrations, the abundance of serum constituents may inhibit the interaction of RBD-conjugated liposomes and immobilized ACE2, resulting in a decrease of signal intensities even in the absence of neutralizing antibodies. Therefore, the ACE2 immobilization strategy was changed from mere adsorption to site-directed binding through a biotin tag on the ACE2 protein and commercially available streptavidin-coated microtiter plates. This led to three improvements of the assay: (i) less ACE2 was needed for the immobilization, hence saving on reagents (1 μg/mL vs. 5 μg/mL), (ii) serum proteins would not adsorb to the surface and hence would not enhance the SRB fluorescence signal in an uncontrolled fashion, and (iii) binding between RBD-conjugated liposomes and ACE2-coated surfaces was improved due to the site-directed immobilization, i.e., 4× higher signals were obtained (Figs. S6–8).

**Fig. 2:**
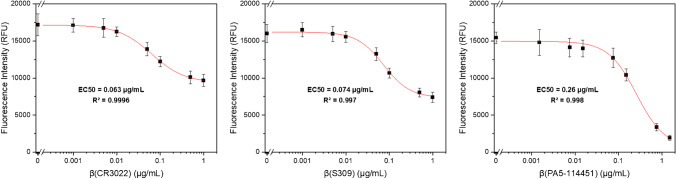
Fluorescence intensities of RBD-conjugated liposomes (50 mM SRB, 1 μM total lipid concentration) tested with binding (CR3022 and S309) and neutralizing (PA5-114451) anti-RBD antibodies (0 to 1 μg/mL). *n* = 3. Curves were fitted using Origin 2022’s Logistic function

With these optimized conditions, the antibody titration curves showed complete prevention of liposome binding by the RBD specific neutralizing antibodies (PA5-114451), while monoclonal antibodies directed against the CR3022 cryptic site and the S309 proteoglycan site caused only partial inhibition (Fig. 2). This suggests that the polyclonal PA5-114451 antibodies fully block binding of the RBD decorated liposomes to the immobilized ACE2 receptor, presumably due to antibodies that are binding within the receptor-binding motif and therefore outcompete ACE2 (class 1 and 2 antibodies) [35]. The S309 (class 3) and CR3022 (class 4) antibodies are not binding within the receptor-binding motif of RBD [35, 36]. S309 is a neutralizing antibody that binds distal from the receptor-binding motif and has neutralizing capabilities in vitro [37]. CR3022 is described as a binding but non-neutralizing antibody, since its epitope is not accessible in trimeric spike protein due to masking by the neighboring protomer [38, 39]. Moreover, both epitopes are not affected by N501Y mutation of B1.1.7 [40]. However, in this assay, monomeric RBD B1.1.7 is employed where the CR3022 epitope is fully accessible for binding antibodies. Thus, CR3022 might exhibit apparent inhibitory activity against the now artificially exposed epitope. Our results may suggest that both antibodies seem to cause steric hindrance for the interaction of the liposomes with the immobilized ACE2 but are not preventing it, resulting in partial inhibition, since both antibodies only bind allosteric to the receptor-binding motif.

**Fig. 3:**
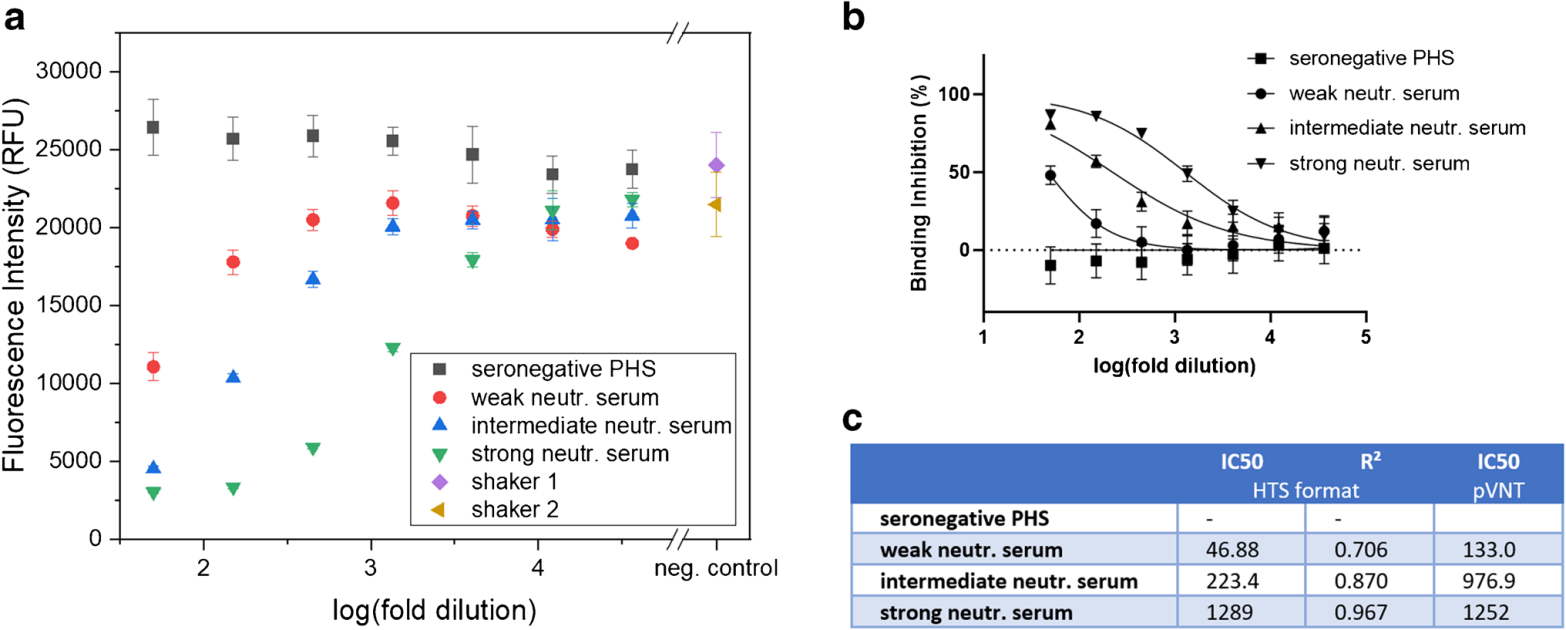
Fluorescence intensities (**a**), binding inhibition curves (**b**), and IC50 values determined via GraphPad Prism 9 using the “log(inhibitor) vs. normalized response with variable slope” algorithm (**c**) of RBD-conjugated liposomes (50 mM SRB, 1 μM total lipid concentration) tested with seronegative pooled human serum (PHS), a weakly, intermediate, and a strongly neutralizing serum (2 v% followed by 3-fold series dilution). *n* = 3. Seronegative PHS and strong neutr. serum samples were incubated at 30 °C and 300 rpm in shaker 1, intermediate and weak neutr. serum in shaker 2. IC50 values determined for the weak, intermediate, and strong neutralizing sera in the pVNT are listed in **c** for comparison

For proof of principle, few carefully selected antisera — a seronegative pooled human serum (PHS), and three sera with known weak, intermediate, and strong neutralizing activity — were used to gather initial data regarding liposome binding inhibition (Fig. 3). In all cases, IC50 values for the neutralization could be determined, which overall correlated with the neutralization activity determined previously via a pseudovirus neutralization test [32] (Fig. 3c). However, the IC50 values for the weak and intermediate neutralizing sera were lower in the HTS format compared to the pVNT and the *R*^2^ value decreased below 0.8 for the weakly neutralizing serum, which suggests that higher serum concentrations would be required for a reliable determination of the IC50 value of sera with low nAb titers. Interestingly, a slight enhancement of the fluorescent signals could still be observed with the seronegative sample (a pooled, commercially available serum) with increasing serum concentration, which was also seen with other samples, mainly seronegative ones from donors with other respiratory diseases (Fig. S9). This suggests that some sera may indeed cause non-specific binding to the microtiter plate surface albeit its blocked surface, and hence lead to a small increase in fluorescence. However, this effect has no impact on the determination of the IC50 values as the resulting negative binding inhibition values are counted as 0% binding inhibition for the analysis.

**Fig. 4:**
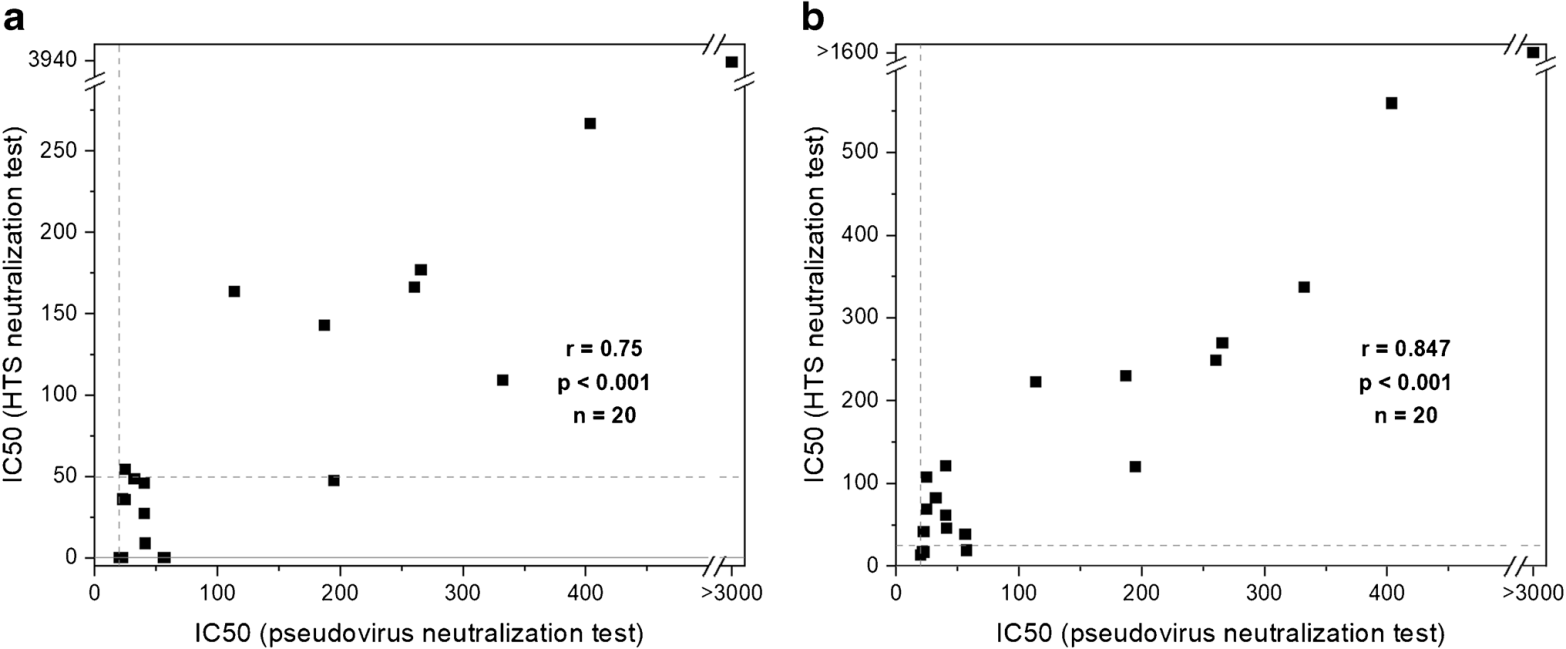
Correlation of IC50 values for 20 neutralizing sera (TiKoCo-19) obtained with a standard pseudovirus neutralization test [32] with IC50 values obtained with the new liposome HTS neutralization test. **a** 50 mM SRB liposomes, 1 μM total lipid concentration, 2% serum followed by 3-fold series dilution and **b** 150 mM SRB liposomes, 0.5 μM total lipid concentration, 4% serum followed by 2-fold series dilution. *n* = 3. The lowest dilution factors used in the neutralization tests are displayed as dashed grey lines. A Spearman correlation was conducted for the 20 samples in **a** (*r* = 0.75, *p* < 0.001) and **b** (*r* = 0.847, *p* < 0.001) using SPSS

Finally, the liposome-based assay was challenged with a serum panel of 20 neutralizing sera, ranging from weak to very strong neutralization as determined by an established pseudovirus neutralization test [32]. It could be seen that the liposome assay had a good correlation with the established test (Spearman’s *r* = 0.75, Fig. 4a); however, the *R*^2^ values were in some instances too low (Table S2). Thus, higher serum concentrations and liposomes encapsulating 150 mM SRB were investigated. The higher fluorescence intensities obtainable with these liposomes enabled the reduction of liposome concentration from 1 to 0.5 μM and hence an improvement in sensitivity. IC50 values could be determined for all sera (Table S2, Fig. S10). Reliable IC50 values could be obtained for 15 out of the 20 sera. The other 5 showed values outside of the investigated range of serum dilutions (1:25 to 1:1600) so that low IC50 values correlated to low *R*^2^ values. Strong correlation was observed between the IC50 values determined with the HTS and pseudovirus neutralization assay (Spearman’s *r* = 0.847, Fig. 4b). Additionally, 10 prepandemic sera were analyzed using the optimized assay conditions. No neutralization was observed for any of the samples despite the increased serum concentrations (Fig. S11), hinting at high specificity of the assay. In the future, further improvement of the sensitivity can be achieved using even higher serum concentrations or by optimization of the RBD coverage regarding proper orientation. Extensive screening including hundreds of seronegative and neutralizing samples could then enable validation of the developed neutralization test toward product development.

### Point-of-care format (POC)

In addition to the development of the high-throughput format, a POC test based on a lateral flow assay was investigated. 150 mM SRB encapsulating liposomes were used to establish a robust POC test. The POC assay format is vastly simplified in contrast to the HTS format resulting in a 1- or 2-step assay. Here, RBD-conjugated liposomes are incubated with serum. Subsequently, biotinylated ACE2 is added to the solution and the mixture is applied to an LFA membrane containing streptavidin in the test line. Hence, RBD-conjugated liposomes bound to ACE2 will collect at the test line, so that the presence of neutralizing antibodies results in lower to no signal (Fig. 1). In initial experiments, liposome concentration (Fig. S12), ACE2-biotin concentration (Fig. S13), and incubation times (Fig. S14) were optimized. The final protocol included 10 μM liposomes, 3× ACE2-biotin concentration in relation to RBD concentration per sample, and no required incubation of ACE2-biotin with the liposome-serum mixture. Furthermore, a control line was developed using immobilized anti-FITC antibody and FITC-conjugated SRB liposomes. It was found that a concentration of 12.9 μM FITC-conjugated liposomes produced the same signal intensity as 10 μM RBD-conjugated liposomes in presence of seronegative pooled human serum (Fig. S15). Under these conditions, mean binding inhibition of −2 ± 3% for the seronegative serum was observed, proving the reproducibility of the format (Fig. S16).

**Fig. 5:**
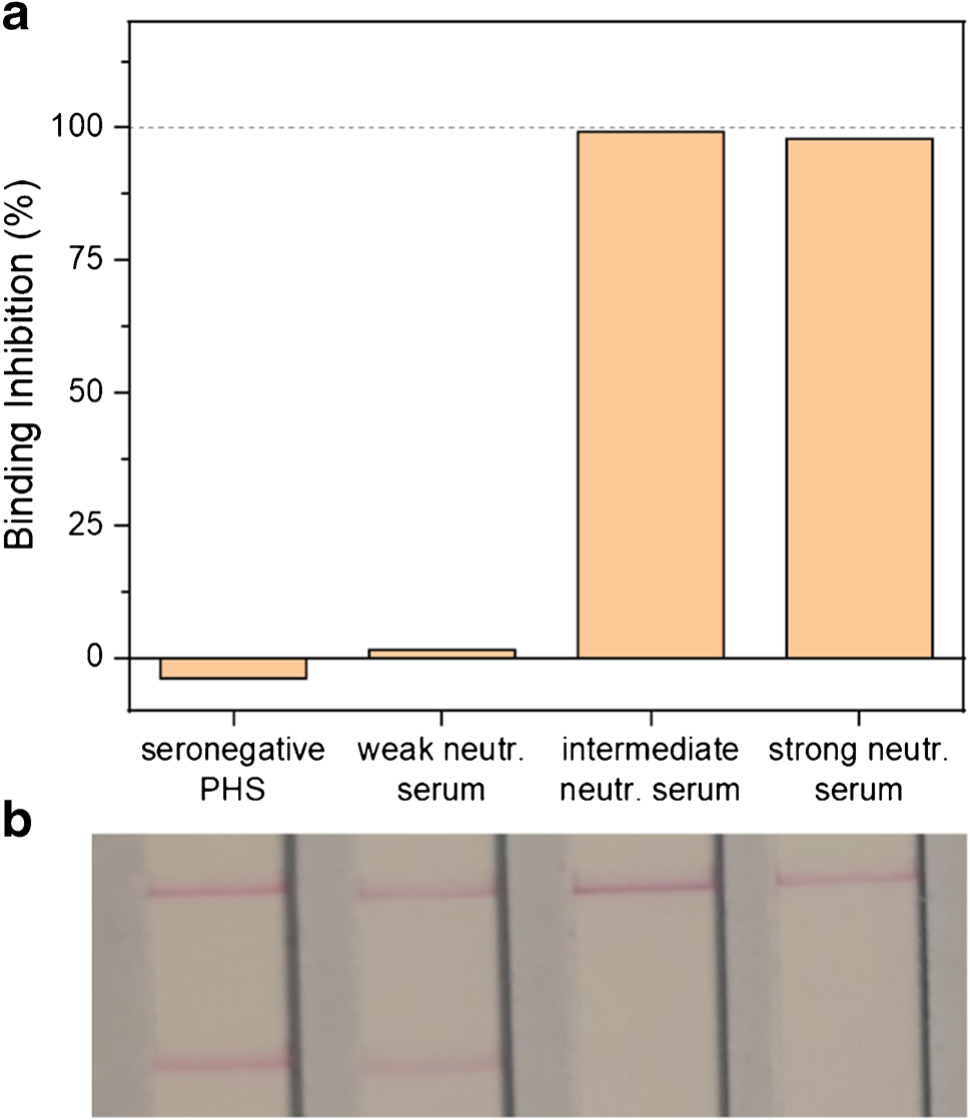
Proof-of-principle assay for the POC format using RBD-conjugated liposomes (150 mM SRB) and ACE2-biotin applied to LFAs with a streptavidin test line and an anti-FITC control line. Test strips (**a**) and binding inhibition values (**b**) of seronegative pooled human serum (PHS), a weakly, intermediate, and a strongly neutralizing serum are shown. *n* = 1

In a proof-of-principle test, 4 sera were analyzed and demonstrated that with the current format, weakly and strongly neutralizing sera could easily be distinguished (Fig. 5). Complete binding inhibition was observed for the intermediate and strongly neutralizing human sera, while no signal decrease was observed for the weakly neutralizing serum. In an attempt for further simplification of the assay protocol, ACE2-biotin addition before or after pre-incubation of liposomes with serum using the same sera proved better performance of the latter (Fig. S17). While this results in an additional pipetting step after the pre-incubation, the added sensitivity may in the end be beneficial.

Furthermore, to reduce the overall assay time, the pre-incubation of liposomes with serum was investigated between 0 min, 5 min, and 15 min (Fig. S18). Not surprisingly, the longer the incubation, the more binding inhibition can be observed. However, the effect was not too pronounced, making 5 min pre-incubation feasible. Nonetheless, an incubation time of 15 min was chosen for all studies in this work to gain optimum sensitivity and ease simultaneous handling of large quantities of test strips. A washing step was also added to reach optimum sensitivity, causing a slight signal increase, but is not crucial, background intensities remaining identical with and without it (data not shown).

Further assay optimizations included avoidance of non-specific binding of liposomes to the LFA membrane strip as this would directly affect signal read-out in the test line. This might i.e. result in fewer liposomes binding to the test or control line and therefore be interpreted as false-positive or false-negative signal. This was already observed for the control liposomes with the weakly neutralizing serum (Fig. S17) and confirmed for 5 out of 24 sera, which were well characterized and carefully selected from the Munich Cohort serum panel (Suppl. Table S3; comprising (i) prepandemic sera, (ii) SARS-CoV-2 reconvalescent sera, (iii) sera obtained from vaccinated individuals, (iv) reconvalescent sera following infection with respiratory disease viruses other than SARS-CoV-2, and (v) sera from previously mycoplasma infected individuals known to be cross-reactive). Noteworthy, this effect was most pronounced for 2 sera with known previous mycoplasma infection (Fig. S19). Using a membrane with larger pore sizes significantly reduced this non-specific binding (Fig. S20). This suggests that these sera caused an agglomeration of liposomes too large to pass through the nitrocellulose pores. Additionally, the investigated sera highlighted the different behavior of seronegative samples and those from convalescent and vaccinated donors in the assay. They had previously been analyzed for RBD-binding antibodies in the *recom*Line SARS-CoV-2 IgG assay. Screening of the sera showed that high binding antibody titers (>500 BAU/mL) corresponded to strong binding inhibition in the POC test. Complete binding inhibition was observed for all 5 sera with high titers (Fig. S19). For intermediate titers between 100 and 200 BAU/mL, no correlation between signal decrease and antibody titer was observed. Seronegative samples expectedly showed no binding inhibition, while in some cases, a reduced control line intensity even resulted in negative binding inhibition values.

To determine the background threshold, six seronegative samples were tested in triplicates, resulting in binding inhibition values around 0% in most cases, with exception of blood donations 4498 and 4500 with values of 10 ± 7% and −10 ± 3%, respectively (Fig. S21). The average of all 18 measurements (0%) + 3× the standard deviation (11%) was used as preliminary cut-off (33%) for further experiments. Additional 10 seronegative samples were tested to verify the accuracy of the cut-off. All sera produced binding inhibition values <0% (Fig. S22), hinting at good specificity of the assay. However, two sera showed exceptionally low values of −24% and −53% (blood donations 4505 and 4507) due to reduced control line intensities. No non-specific binding was observed, suggesting that serum constituents affected the interaction of FITC-modified liposomes with the immobilized anti-FITC antibodies.

**Fig. 6:**
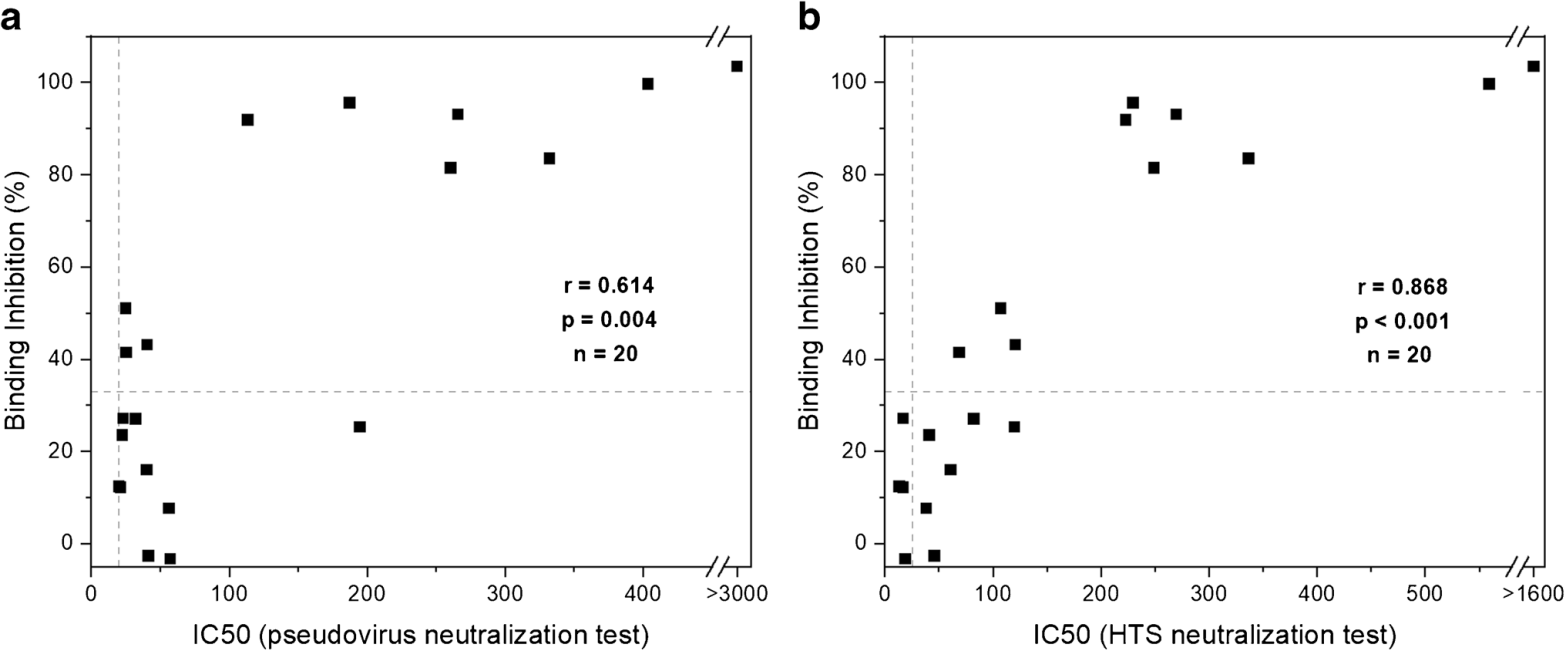
Correlation of binding inhibition values determined in the POC neutralization test and IC50 values determined in the pseudovirus (**a**) and the HTS neutralization test (**b**) of 20 neutralizing sera (TiKoCo-19). Spearman correlation coefficients of *r* = 0.614 (**a**) and *r* = 0.868 (**b**) were calculated using SPSS. The lowest dilution factors used in the neutralization tests and the preliminary cut-off calculated for the POC test are displayed as dashed grey lines

Following the above assay optimization and determination of background threshold, the identical serum panel (*n* = 20), which was already used to validate the HTS assay format (Fig. 3), was analyzed with the optimized POC assay (Fig. S23, Table S2). Complete binding inhibition of >95% was observed in the POC format for 3 out of the 20 sera. Another 4 sera showed high values above 80%, while the remaining 13 sera showed values <60%. A total of 10 sera gave values below the established threshold of 33%. Correlation of these binding inhibition values to the IC50 values determined with the pVNT showed a Spearman’s *r* of 0.614 (Fig. 6a). Despite the mediocre quantitative correlation, those results still prove sufficient qualitative detection of neutralizing antibodies by the POC assay. Specifically, all but one serum with IC50 values >100 show >80% binding inhibition, while sera with IC50 values between 20 and 70 showed anywhere between 0 and 50% binding inhibition. Furthermore, very good correlation was observed for the two liposome-based formats with a Spearman’s *r* of 0.868 (Fig. 6b). IC50 values >200 correlated close to complete binding inhibition, values <50 corresponded to 0–30% binding inhibition. As the POC format is significantly faster and simpler to perform, it leads to a qualitative nature-only at this point, which will be improved upon in the future. Here, better sensitivity and hence actually quantification can be achieved by increasing the liposome diameter [25], entrapping other dyes or investigation of directed protein immobilization on the liposome surface.

### Liposome stability

Liposomes without any proteins coupled to their surface or those coupled to streptavidin have previously been shown to be highly stable when stored at 4 °C for years [25]. In contrast, RBD-conjugated liposomes were found to be stable for only 1 month at 4 °C (Fig. S24). Longer storage times led to agglomeration and precipitation of the liposomes. Thus, in the future, a formulation of the RBD-liposome buffer will be studied by adding stabilizing agents to avoid the protein-mediated aggregation. Alternatively, streptavidin-coupled liposomes can be used in combination with biotinylated RBD. From a commercial viewpoint, this approach has the advantage that the competitive assays could be quickly adapted for other analytes using different biotinylated proteins, which could be stored separately.

## Conclusion

The developed SRB liposomes enable both the sensitive detection and quantification of neutralizing antibodies against RBD in a microplate-based high-throughput assay as well as the qualitative detection in a lateral flow-based POC format. Very good correlation was observed between the two formats, while only the HTS format showed strong correlation to an established pseudovirus neutralization test (pVNT), confirming the ability of the assay to detect neutralizing antibodies in the desired range. The HTS format could determine IC50 values within the investigated range for 15 out of the 20 sera tested with the pseudovirus neutralization test. Further improvement of the sensitivity could be achieved with the use of higher serum concentrations or the use of different liposome species, e.g., chemiluminescent or HRP liposomes, enabling reliable IC50 determination even for weakly neutralizing sera. Another option may be the optimization of RBD coverage regarding neutralization, and lower coverage could improve the sensitivity at the expense of signal intensity. The advantage over currently used ELISA-type HTS formats is the quick and not-time-dependent signal read-out afforded by the liposomes. In contrast to the enzyme reaction-based read-out strategies, more reproducible and simpler HTS strategies can therefore be achieved. The POC format enables fast detection of neutralizing antibodies, albeit only in a qualitative manner at this point. Nevertheless, extrapolation with population-based data [41] would support a quantitative read-out, as the majority of the population’s titers (vaccinated or recently infected) should be in the assay’s dynamic range. The use of a membrane with larger pore size might improve the correlation to the pVNT and enable a semi-quantitative determination of nAbs. Implementation of a signal on strategy by addition of a secondary test line consisting of anti-human IgG antibodies to capture neutralized liposomes might also improve the sensitivity of the assay, as shown in literature [19, 24]. Additionally, this setup might make the test more intuitive for users, stronger test line intensities correlating to higher neutralizing antibody titers. Optimization of the assay, i.e., shortening of pre-incubation time and omitting of the washing step, would allow for assessment of the immune status of a patient within <30 min after blood-draw.

Both test formats demonstrate that even though liposomes are significantly larger than gold nanoparticles (20–80 nm) and enzymes (<10 nm), their multivalency overcomes possible steric hindrance in the competitive assay format and hence enable the development of highly effective and reliable HTS and POC diagnostic tests. Furthermore, since the signaling molecules are hidden from the assay itself by being inside the liposomes, several unique characteristics are afforded by liposomes. Specifically, entrapping different dyes can easily prepare liposomes for multianalyte strategies, and control lines can be made with e.g. blue or green rather than magenta liposomes. Also, the concentration range of an analyte that is covered within the dynamic range of the assay can easily be adapted by using different dye concentrations encapsulated within the liposomes. The same can be achieved by simply changing the liposomes diameter [25]. The entrapment of other marker molecules inside the liposomes even broadens read-out capabilities far beyond fluorescence and colorimetric approaches and have been demonstrated with chemiluminescent, bioluminescent, electrochemical, and electrochemiluminescent detection strategies before. Finally, in contrast to most other signaling agents, the chemical surface of liposomes can easily be adapted to a specific coupling chemistry or matrix without the need for complex reactions. The addition of lipids with varying head groups instead easily establishes alternative chemistries, such as biotinylation, amine groups, click chemistry, or ganglioside receptors. These properties make liposomes a versatile tool for a multitude of assays, including competitive binding assays, such as neutralization tests, as demonstrated in this work.

## Supplementary Information

ESM 1 (DOCX 3.48 MB)
