# Supplementary material for: Liposome-based high-throughput and point-of-care assays toward the quick, simple, and sensitive detection of neutralizing antibodies against SARS-CoV-2 in patient sera
Source: Anal Bioanal Chem. 2023 Feb 9;415(8):1421–35. doi: 10.1007/s00216-023-04548-3 (PMC9909147; doi:10.1007/s00216-023-04548-3)
Supplement: Supplementary file 1 — (DOCX 3.48 MB) [file 216_2023_4548_MOESM1_ESM.docx]

**Electronic Supporting Material**

**Liposome-based high-throughput and point-of-care assays toward the quick, simple and sensitive detection of neutralizing antibodies against SARS-CoV-2 in patient sera**

Simon Streif^1^, Patrick Neckermann^2^, Clemens Spitzenberg^1^, Katharina Weiss^1^, Kilian Hoecherl^1^, Kacper Kulikowski^1^, Sonja Hahner^3^, Christina Noelting^3^, Sebastian Einhauser^2^, David Peterhoff^2,4^, Claudia Asam^2^, Ralf Wagner^2,4^, Antje J. Baeumner^1*^

^1^ Institute of Analytical Chemistry, Chemo‑ and Biosensors, University of Regensburg, Universitaetsstr. 31, 93053 Regensburg, Germany

^2^ Institute of Medical Microbiology & Hygiene, Molecular Microbiology (Virology), University of Regensburg, Universitaetsstr. 31, 93053 Regensburg, Germany

^3^ Mikrogen GmbH, Floriansbogen 2-4, 82061 Neuried, Germany

^4^ Institute of Clinical Microbiology and Hygiene, University Hospital Regensburg

***Author for correspondence:**

**Email: antje.baeumner@ur.de**

**Phone: +49 941 943 4065**

**Postal address:**

**Antje Baeumner**

**Universität Regensburg**

**93040 Regensburg**

**Germany**

**Outline**

[1 Experimental section 2](#_Toc119401720)

[1.1 Chemicals and consumables 2](#_Toc119401721)

[1.2 Buffer compositions 2](#_Toc119401722)

[1.3 Liposome composition 2](#_Toc119401723)

[2 Results 3](#_Toc119401724)

[2.1 Quality control of recombinant proteins 3](#_Toc119401725)

[2.2 High-throughput format (HTS) 4](#_Toc119401726)

[2.2.1 Assay development 4](#_Toc119401727)

[2.2.2 Serum panel screening 8](#_Toc119401728)

[2.3 Point-of-care format (POC) 11](#_Toc119401729)

[2.4 Liposome stability 20](#_Toc119401730)

[References 20](#_Toc119401731)

# 1 Experimental section

## 1.1 Chemicals and consumables

Sucrose, sodium azide, sodium chloride and dialysis membrane Spectra/Por© 4 (MWCO: 12-14 kDa) (2718.1) were purchased from Carl Roth. Phosphorous standard was obtained from Bernd Kraft GmbH (Germany). Chloroform, methanol and Spectra-Por® Float-A-Lyzer® G2 (1 mL, MWCO: 1000 kDa) were purchased from Fisher Scientific. Whatman Nucleopore™ Track-Etched membranes (1.0 µm, 0.4 µm and 0.2 µm diameter) and Tween® 20 were obtained from Sigma Aldrich/Merck (Germany).

## 1.2 Buffer compositions

High sucrose saline (HSS) buffer contained 200 mM sucrose, 200 mM NaCl, 10 mM HEPES and 0.01 w% NaN_3_, pH 7.5. PMB contained 10 mM HEPES, 150 mM NaCl, 135 nM CaCl_2_ and 1 mM MgCl_2_, pH 7.4. PBS contained 137 mM NaCl, 2.7 mM KCl, 10 mM Na_2_HPO_4_ and 1.8 mM KH_2_PO_4_, pH 7.4. PBS-T contained 0.1 w% Tween^®^ 20 in PBS.

## 1.3 Liposome composition

**Table S 1** Lipid and encapsulant compositions of all used liposomes.

|  | lipids | | | | | encapsulant | | |  |
| --- | --- | --- | --- | --- | --- | --- | --- | --- | --- |
|  | cholesterol  /mg | DPPC  /mg | DPPG  /mg | DMPE  /mg | N-glut.-DPPE  /mg | | SRB  /mg | NaCl  /mg | |
| 10 mM SRB COOH-liposomes | 2.4 | 66.4 | 17.9 | - | 4.2 | | 26.7 | 55.0 | |
| 50 mM SRB COOH-liposomes | 9.8 | 14.4 | 8.5 | - | 4.0 | | 126.3 | 52.9 | |
| 150 mM SRB COOH-liposomes | 9.6 | 14.2 | 8.2 | - | 3.9 | | 376.8 | 36.9 | |
| 100 mM SRB  NH_2_-liposomes | 10.0 | 14.9 | 8.8 | 2.0 | - | | 252.2 | 53.6 | |
|  |  |  |  |  |  | |  |  | |

# 2 Results

## 2.1 Quality control of recombinant proteins

Purity of recombinant proteins was proven using reducing SDS-PAGE. Only the protein of interest could be observed (Fig. S 1 A and B). To analyze correct folding and biotinylation of RBD and ACE2, an ELISA experiment was performed using coated RBD on the solid phase and titration of biotinylated ACE2 compared to non-biotinylated ACE2. The ELISA revealed rigid binding of biotinylated ACE2 with a dissociation constant of 5.4 nM ± 3 (Fig. S 1 C).


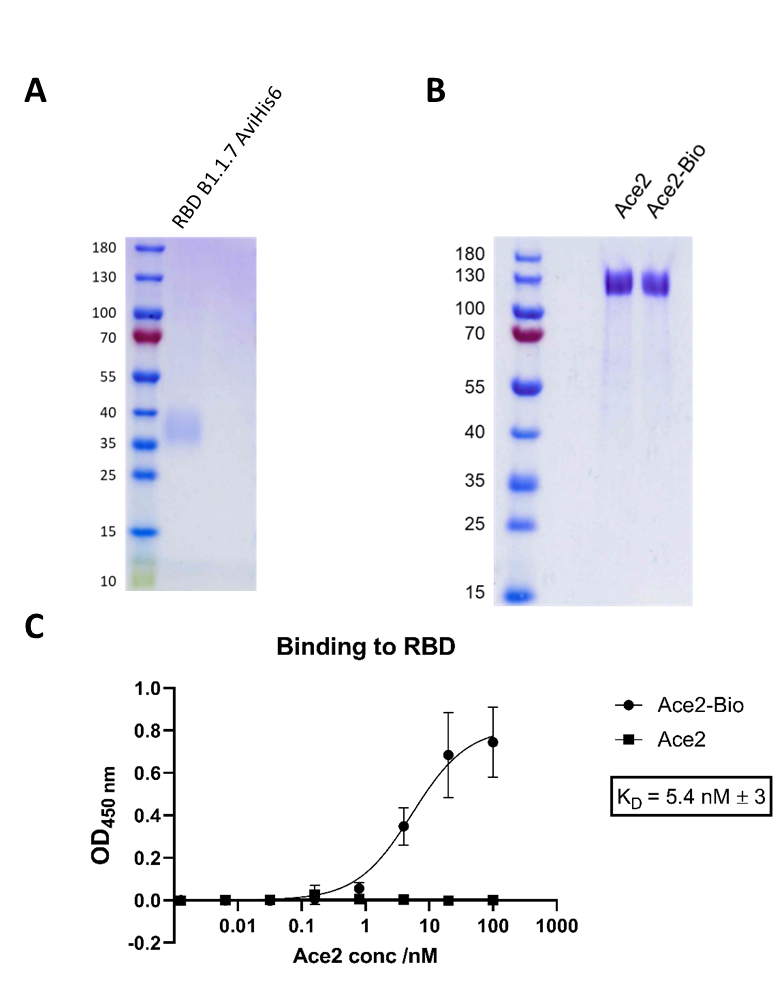


**Fig. S 1** Quality control of recombinant proteins. Reducing SDS-PAGE of RBD B1.1.7 (**A**) and biotinylated and non-biotinylated ACE2 (**B**). 2 µg protein were loaded per lane. (**C**): Binding of biotinylated and non-biotinylated ACE2 against RBD B1.1.7 measured by ELISA. Values were fitted using non-linear curve regression (one-side hyperbola fit). n = 3.

## 2.2 High-throughput format (HTS)

### 2.2.1 Assay development

Different RBD concentrations were conjugated to 10 mM SRB liposomes bearing 4 mol% lipids with carboxylated headgroups via EDC/NHS chemistry. Immobilization in an ACE2-coated microplate showed that 0.2 mol% RBD performed best. A concentration of 0.5 mol% resulted in decreased liposome immobilization, either due to dense packing or cross-linking of RBD, causing steric hindrance for the RBD-ACE2 interaction. Investigation of 0.3 mol% and 0.4 mol% showed no improvement compared to the 0.2 mol% RBD (data not shown).

**Fig. S 2** Fluorescence intensities of 10 mM SRB encapsulating liposomes conjugated to 0.1 mol%, 0.2 mol% and 0.5 mol% RBD (10 µM total lipid concentration) immobilized in a Nunc MaxiSorp high binding microplate coated with 0 or 5 µg/mL ACE2 in PBS (100 µL). The plate was previously blocked with 1 w/v% BSA in PBS-T (150 µL) and washed with PBS-T (2x, 150 µL) and PMB (3x, 150 µL). Samples were incubated for 3h at RT and 300 rpm, washed with PMB (3x, 150 µL) and lysed by addition of 30 mM OG in bidest. H_2_O (100 µL, 10 min inc., RT, 300 rpm). The fluorescence was measured using a BioTek SYNERGY neo2 fluorescence reader (𝜆_𝐸𝑥_ = 560 𝑛𝑚 and 𝜆_𝐸𝑚_ = 585 𝑛𝑚, bandwidth 10, gain 150). n = 3.

Investigation of ACE2-coating showed that higher concentrations produced better signal intensities. A concentration of 5 µg/mL ACE2 was chosen for coating. No higher concentrations were investigated to safe on reagents.

**Fig. S 3** Fluorescence intensities of 10 mM SRB encapsulating liposomes conjugated to 0.2 mol% RBD (5 µM and 10 µM total lipid concentration) immobilized in a Nunc MaxiSorp high binding microplate coated with 0, 1, 2 or 5 µg/mL ACE2 in PBS (100 µL). The plate was previously blocked with 1 w/v% BSA in PBS-T (150 µL) and washed with PBS-T (2x, 150 µL) and PMB (3x, 150 µL). Samples were incubated for 3h at RT and 300 rpm, washed with PMB (3x, 150 µL) and lysed by addition of 30 mM OG in bidest. H_2_O (100 µL, 10 min inc., RT, 300 rpm). The fluorescence was measured using a BioTek SYNERGY neo2 fluorescence reader (𝜆_𝐸𝑥_ = 560 𝑛𝑚 and 𝜆_𝐸𝑚_ = 585 𝑛𝑚, bandwidth 10, gain 150). n = 3.

A titration curve of 50 mM SRB liposomes conjugated to 0.2 mol% RBD showed a linear correlation of fluorescence intensity and liposome concentration between the investigated 0.1 µM and 10 µM. Concentrations of 0.5 µM and 1 µM were chosen for further experiments.

**Fig. S 4** Fluorescence intensities of RBD-conjugated liposomes in PMB (50 mM SRB, 0, 0.1, 0.2, 0.5, 1, 2, 5 and 10 µM total lipid concentration) immobilized in a Nunc MaxiSorp high binding microplate coated with 5 µg/mL ACE2 in PBS (100 µL). The plate was previously blocked with 1 w/v% BSA in PBS-T (150 µL) and washed with PBS-T (2x, 150 µL) and PMB (3x, 150 µL). Samples were incubated for 3h at RT and 300 rpm, washed with PMB (3x, 150 µL) and lysed by addition of 30 mM OG in bidest. H_2_O (100 µL, 10 min inc., RT, 300 rpm). The fluorescence was measured using a BioTek SYNERGY neo2 fluorescence reader (𝜆_𝐸𝑥_ = 560 𝑛𝑚 and 𝜆_𝐸𝑚_ = 585 𝑛𝑚, bandwidth 10, gain 150). n = 3.

Titration curves of antibodies directed against the receptor binding site (PA5-114451) and the CR3022 and S309 binding sites of RBD showed complete inhibition of RBD-ACE2 interaction by the former. CR3022 caused partial inhibition, signals not allowing for a sigmoidal fit. S309 caused a signal decrease of >50%, but did not result in complete inhibition. The lower EC50 value compared to PA5-114451 suggests that the S309 antibody has a higher affinity.


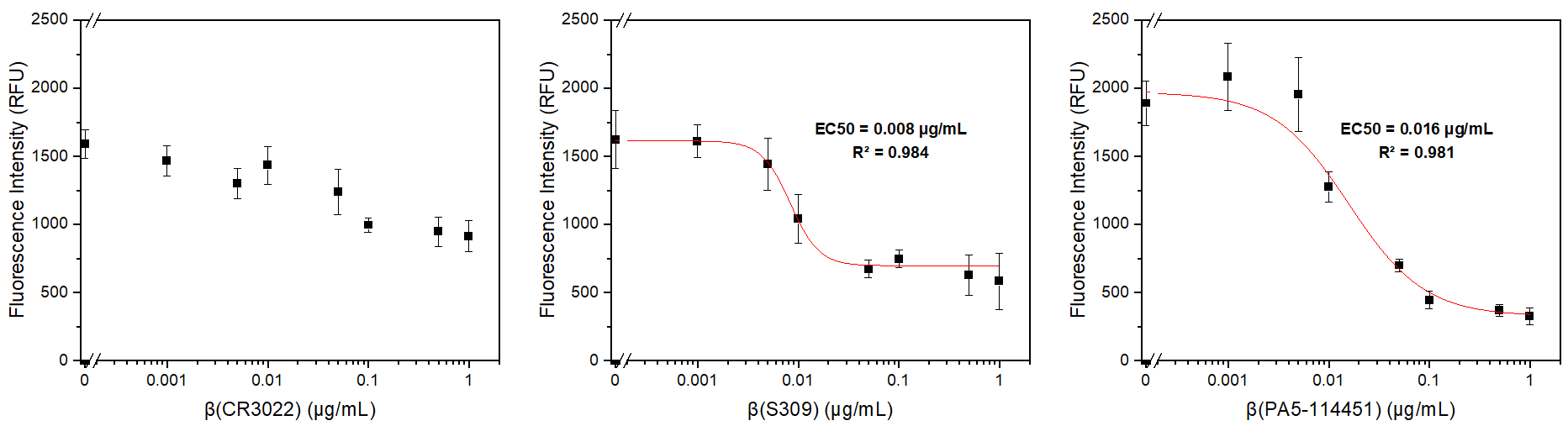


**Fig. S 5** Fluorescence intensities of RBD-conjugated liposomes (50 mM SRB, 0.5 µM total lipid concentration) tested with non‑neutralizing (CR3022 and S309) and neutralizing anti-RBD antibodies (PA5-114451) (0 µg/mL to 1 µg/mL) immobilized (after 1 h pre-incubation at RT, 300 rpm and 32-fold concentration) for 3 h at RT in a Nunc MaxiSorp high binding microplate coated with 5 µg/mL ACE2. After washing with HSS (3x, 150 µL) liposomes were lysed by addition of 30 mM OG in bidest. H_2_O (100 µL, 10 min inc., RT, 300 rpm). The fluorescence was measured using a BioTek SYNERGY neo2 fluorescence reader (𝜆_𝐸𝑥_ = 560 𝑛𝑚 and 𝜆_𝐸𝑚_ = 585 𝑛𝑚, bandwidth 10, gain 150). n = 3. Curves were fitted using Origin’s logistic function.

To improve signal intensities and reduce the effect of serum concentration on the fluorescence intensity directed immobilization of biotinylated ACE2 in a streptavidin-coated microtiter plate was investigated. A 4-fold increase of signal intensities was observed compared to mere adsorption of ACE2 in a Nunc MaxiSorp high binding microtiter plate (Fig. S 6). Further investigations showed that 1 µg/mL ACE2-biotin already produced satisfactory signal intensities, compared to the previously used 5 µg/mL ACE2 (Fig. S 7). Also, no additional blocking step was needed. Comparison of 1-3 h incubation time showed that 2 h resulted in significantly better signals compared to 1 h, while 3 h only caused a minor improvement. An incubation time of 2 h was chosen as compromise for high signal intensities and shorter assay turn-around times. In the end, it was shown that ACE2-biotin immobilization could be accomplished within 1 h at RT and 300 rpm or overnight at 4 °C. The presence of 0.1 v% non-neutralizing serum did not affect signal intensities, unlike previously observed for non-directed ACE2 immobilization (Fig. S 8).

**Fig. S 6** Fluorescence intensities of RBD-conjugated liposomes (50 mM SRB, 1 µM total lipid concentration) immobilized for 3 h at RT in a Nunc MaxiSorp high binding microplate coated with 5 µg/mL ACE2 in PBS (100 µL) and blocked with 1 w/v% BSA in PBS-T (non-directed) or in a streptavidin microplate coated with 5 µg/mL ACE2-biotin in PBS (100 µL) (site-directed). After washing with HSS (3x, 150 µL) liposomes were lysed by addition of 30 mM OG in bidest. H_2_O (100 µL, 10 min inc., RT, 300 rpm). The fluorescence was measured using a BioTek SYNERGY neo2 fluorescence reader (𝜆_𝐸𝑥_ = 560 𝑛𝑚 and 𝜆_𝐸𝑚_ = 585 𝑛𝑚, bandwidth 10, gain 150). n = 4.

**Fig. S 7** Fluorescence intensities of RBD-conjugated liposomes (50 mM SRB, 1 µM total lipid concentration) immobilized for 3 h at RT in a streptavidin microplate coated with 0, 1, 2 and 5 µg/mL ACE2-biotin in PBS (100 µL) unblocked or blocked with 1 w/v% BSA in PBS-T. After washing with HSS (3x, 150 µL) liposomes were lysed by addition of 30 mM OG in bidest. H_2_O (100 µL, 10 min inc., RT, 300 rpm). The fluorescence was measured using a BioTek SYNERGY neo2 fluorescence reader (𝜆_𝐸𝑥_ = 560 𝑛𝑚 and 𝜆_𝐸𝑚_ = 585 𝑛𝑚, bandwidth 10, gain 150). n = 4.

**Fig. S 8** Fluorescence intensities of RBD-conjugated liposomes (50 mM SRB, 1 µM total lipid concentration) with and without 0.1 v% seronegative pooled human serum (IR serum) immobilized for 1 h, 2 h or 3 h at RT in a streptavidin microplate coated with 1 µg/mL ACE2-biotin in PBS (100 µL) overnight at 4 °C or for 1 h at RT and 300 rpm. After washing with HSS (3x, 150 µL) liposomes were lysed by addition of 30 mM OG in bidest. H_2_O (100 µL, 10 min inc., RT, 300 rpm). The fluorescence was measured using a BioTek SYNERGY neo2 fluorescence reader (𝜆_𝐸𝑥_ = 560 𝑛𝑚 and 𝜆_𝐸𝑚_ = 585 𝑛𝑚, bandwidth 10, gain 150). n = 4.

### 2.2.2 Serum panel screening

8 seronegative samples (as determined with the *recom*Line SARS-CoV-2 IgG assay, Table S 3) were analyzed with the HTS neutralization test. As expected, no binding inhibition was observed, hence no IC50 values could be obtained (Fig. S 9). Interestingly, a fluorescence enhancing effect was observed for seronegative samples from donors with other respiratory diseases (RSV, Influenza A, Adenovirus, Mycoplasm).


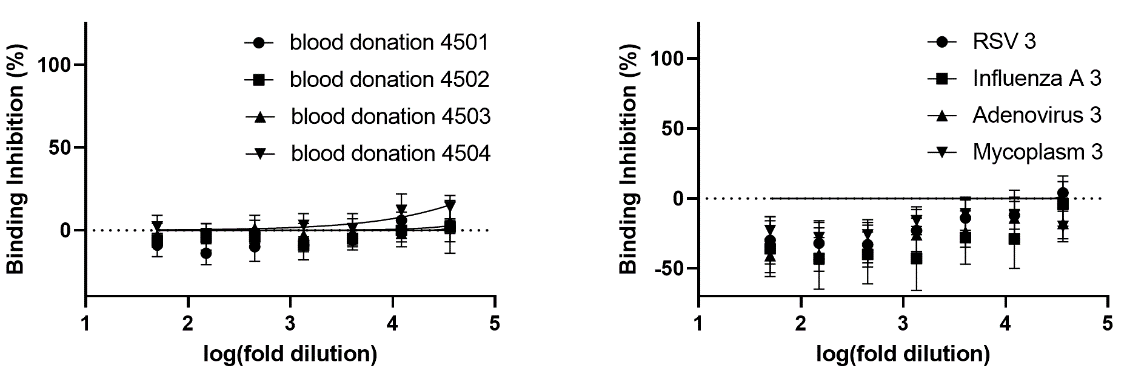


**Fig. S 9** Best-fit graphs for seronegative sera, as determined with the *recom*Line SARS-CoV-2 IgG assay, tested in the HTS neutralization test as calculated using GraphPad Prism 9’s ‘log(inhibitor) vs. normalized response with variable slope’ algorithm. Binding inhibition, given as percentage, was calculated as (1 – fluor. int./fluor. int. neg. control)*100. 2 v% serum, 3-fold series dil. 50 mM SRB lipos. (1 µM).

**Table S 2** Summary of IC50 values of neutralizing sera (nS) tested in the pseudovirus neutralization test and the liposome-based HTS neutralization test. Best-fit IC50 values including R² values were calculated using GraphPad Prism 9 (‘log(inhibitor) vs. normalized response – Variable slope‘ algorithm). IC50 values obtained with the liposome-based HTS neutralization test with R² values <0.9, <0.8 and <0.5 are marked respectively. Additionally, the binding inhibition values obtained in the POC neutralization test are listed.

|  | **pseudovirus**  **neutr. test** | **HTS neutr. test**  2 v% serum, 3-fold series dil.  50 mM SRB lipos. (1 µM) | | **HTS neutr. test**  4 v% serum, 2-fold series dil.  150 mM SRB lipos. (0.5 µM) | | **POC**  **neutr. test** |
| --- | --- | --- | --- | --- | --- | --- |
| **No.** | **IC50** | **IC50** | **R²** | **IC50** | **R²** | **Binding**  **inhibition** |
| nS1 | 21.32 | - | - | 17.09 | 0.6621 | 12% |
| nS2 | 187.4 | 142.9 | 0.9675 | 229.8 | 0.9809 | 96% |
| nS3 | >3000 | 3939 | 0.9909 | 26870 | 0.9052 | 103% |
| nS4 | 57.47 | - | - | 19.00 | 0.7929 | -3% |
| nS5 | 332.3 | 109.2 | 0.9226 | 336.6 | 0.9726 | 83% |
| nS6 | 194.7 | 47.66 | 0.8813 | 119.8 | 0.9310 | 25% |
| nS7 | 40.43 | 27.23 | 0.6254 | 60.89 | 0.8521 | 16% |
| nS8 | 113.7 | 163.4 | 0.9554 | 222.9 | 0.9876 | 92% |
| nS9 | 20 | - | - | 13.21 | 0.7381 | 12% |
| nS10 | 25.21 | 54.30 | 0.8222 | 107.4 | 0.9693 | 51% |
| nS11 | 41.45 | 8.855 | 0.4568 | 45.72 | 0.8881 | -3% |
| nS12 | 260.6 | 166.1 | 0.9490 | 249.1 | 0.9849 | 81% |
| nS13 | 32.41 | 48.64 | 0.7657 | 82.39 | 0.9734 | 27% |
| nS14 | 56.37 | - | - | 38.28 | 0.8988 | 8% |
| nS15 | 403.8 | 266.9 | 0.9719 | 559.5 | 0.9817 | 100% |
| nS16 | 265.9 | 176.8 | 0.9646 | 269.8 | 0.9676 | 93% |
| nS17 | 22.5 | - | - | 41.06 | 0.9044 | 23% |
| nS18 | 25.23 | 35.55 | 0.6064 | 68.74 | 0.9264 | 41% |
| nS19 | 40.5 | 45.83 | 0.8473 | 120.7 | 0.9418 | 43% |
| nS20 | 23.2 | 36.01 | 0.4296 | 16.83 | 0.8637 | 27% |

Table S 2 lists IC50 values for 20 neutralizing sera tested in the pseudovirus neutralization test [1] and the HTS neutralization test (initial format using 50 mM SRB liposomes and optimized format using 150 mM SRB liposomes) as well as binding inhibition values obtained in the POC neutralization test. Binding inhibition curves obtained in the HTS neutralization test are shown in Fig. S 10. Additional 10 seronegative samples were investigated with the optimized conditions (Fig. S 11). No binding inhibition was observed for any of the investigated samples.


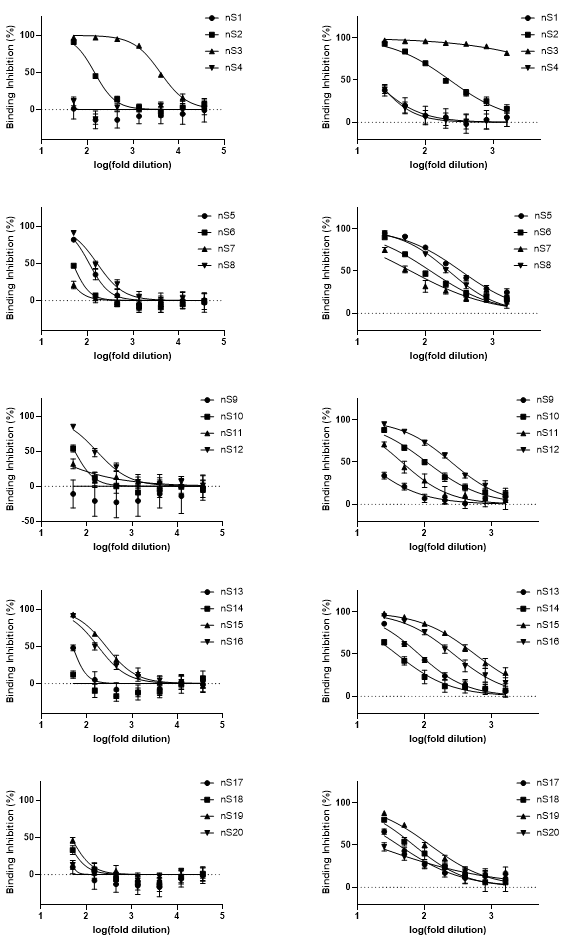


**Fig. S 10** Best-fit graphs for 20 neutralizing sera (nS) tested in the HTS neutralization test as calculated using GraphPad Prism 9’s ‘log(inhibitor) vs. normalized response with variable slope’ algorithm. Binding inhibition, given as percentage, was calculated as (1 – fluor. int./fluor. int. neg. control)*100. Left graphs - 2 v% serum, 3-fold series dil. 50 mM SRB lipos. (1 µM). Right graphs - 4 v% serum, 2-fold series dil. 150 mM SRB lipos. (0.5 µM).


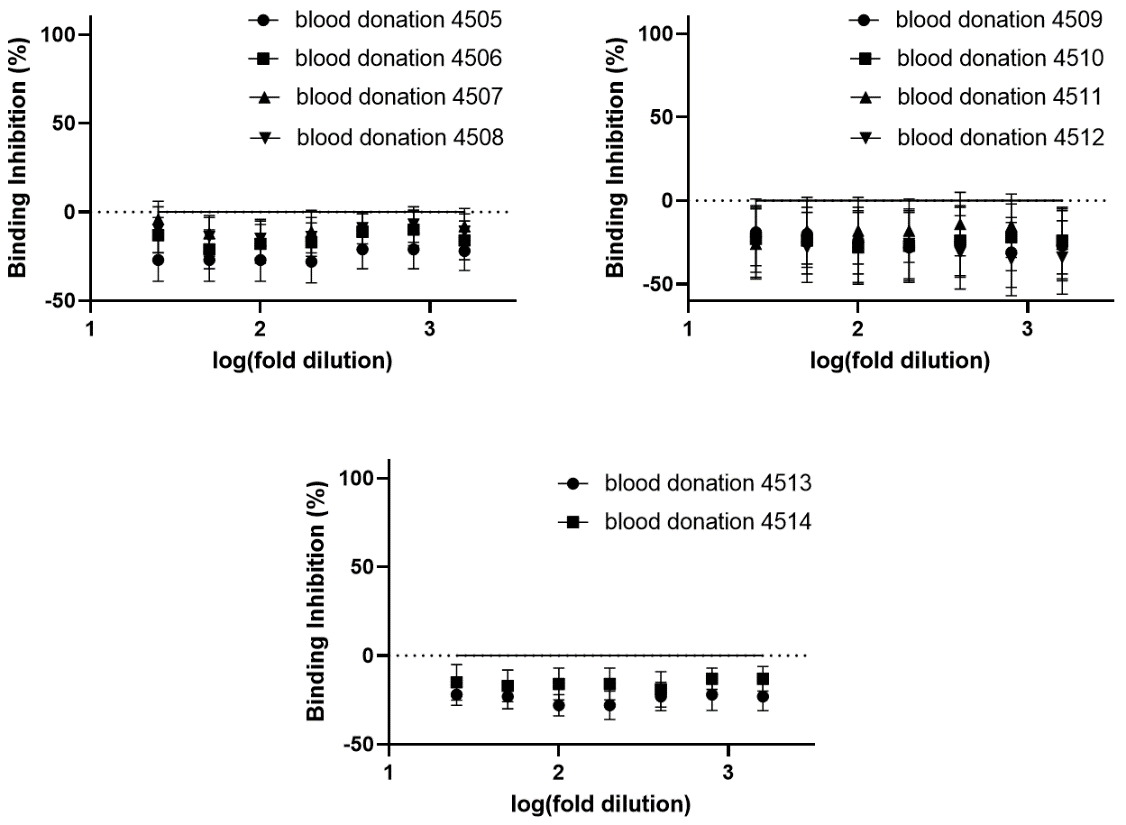


**Fig. S 11** Best-fit graphs for seronegative sera, as determined with the *recom*Line SARS-CoV-2 IgG assay, tested in the HTS neutralization test as calculated using GraphPad Prism 9’s ‘log(inhibitor) vs. normalized response with variable slope’ algorithm. Binding inhibition, given as percentage, was calculated as (1 – fluor. int./fluor. int. neg. control)*100. 4 v% serum, 2-fold series dil. 150 mM SRB lipos. (0.5 µM).

## 2.3 Point-of-care format (POC)

The use of 150 mM SRB liposomes conjugated to 0.2 mol% RBD with 1 equivalent ACE2-biotin and 10 v% non-neutralizing serum on test strips with streptavidin test line was investigated. A linear correlation of signal intensity and liposome concentration was observed between the investigated 5 µM and 20 µM (Fig. S 12). For further studies a concentration of 10 µM was chosen as a compromise between low liposome concentration for a highly sensitive assay and a well visible signal.


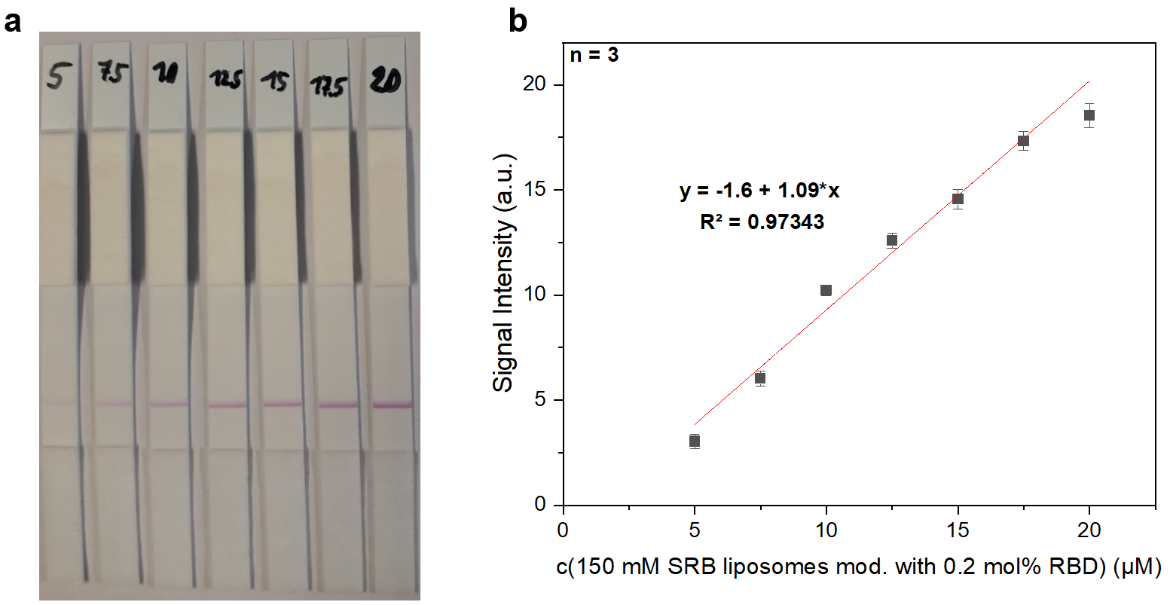


**Fig. S 12** Test strips (a) and their signal intensities (b) of RBD-conjugated liposomes (150 mM SRB, 5-20 µM total lipid concentration) tested with seronegative pooled human serum (10 v%). Samples were pre-incubated for 15 min at RT and 300 rpm in HSS. ACE2-biotin (1 equivalent per RBD) was added to samples before addition to the test strip. Samples were washed after 5 min (25 µL HSS) and pictures were taken after another 20 min using a Canon EOS 550D camera with a Canon EFS 18-55mm lens. Images were analyzed using ImageJ. n = 3.

Investigation of ACE2-biotin concentration showed no improvement for 2 or more equivalents of ACE2-biotin per RBD molecule (Fig. S 13). Repetition of the study showed optimum signal intensity using 3 equivalents (data not shown). This concentration was hence chosen for all further studies. No additional incubation step was needed after addition of ACE2-biotin to the pre-incubated liposome-serum mixture (Fig. S 14).


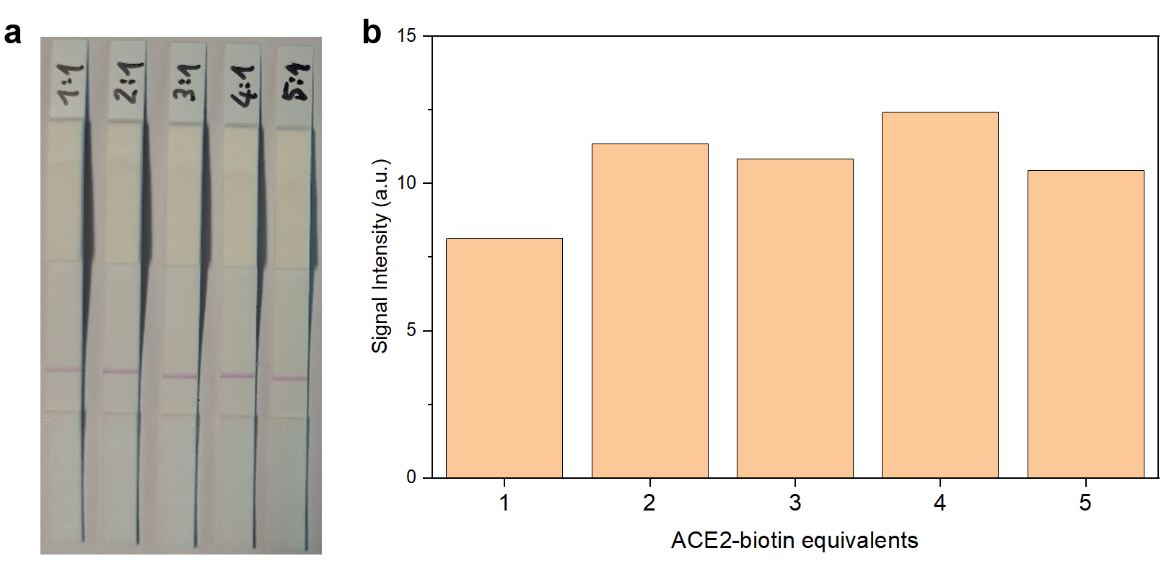


**Fig. S 13** Test strips (a) and their signal intensities (b) of RBD-conjugated liposomes (150 mM SRB, 10 µM total lipid concentration) tested with seronegative pooled human serum (10 v%). Samples were pre-incubated for 15 min at RT and 300 rpm in HSS. ACE2-biotin (1, 2, 3, 4 or 5 equivalents per RBD) was added to samples before addition to the test strip. Samples were washed after 5 min (25 µL HSS) and pictures were taken after another 20 min using a Canon EOS 550D camera with a Canon EFS 18-55mm lens. Images were analyzed using ImageJ. n = 1.

**Fig. S 14** Signal intensities of RBD-conjugated liposomes (150 mM SRB, 10 µM total lipid concentration) tested with seronegative pooled human serum (10 v%). Samples were pre-incubated for 15 min at RT and 300 rpm in HSS. ACE2-biotin (3 equivalents per RBD) was added to samples and a second incubation step (0-60 min) added before addition to the test strip. Samples were washed after 5 min (25 µL HSS) and pictures were taken after another 20 min using a Canon EOS 550D camera with a Canon EFS 18-55mm lens. Images were analyzed using ImageJ. n = 1.

Liposomes conjugated with fluorescein 5(6)-isothiocyanate (FITC) were developed to serve as a control that could be captured on an <anti-FITC> line on the nitrocellulose membrane. FITC was coupled to amine groups on the liposomal surface in carbonate buffer (100 mM NaHCO_3_, 250 mM NaCl, pH 9) overnight followed by dialysis. A linear range of signal intensity to liposome concentration was observed between 3 µM and 16.5 µM and a concentration of 12.9 µM FITC-conjugated liposomes was shown to produce an identical signal intensity as 10 µM RBD-conjugated liposomes (Fig. S 15).


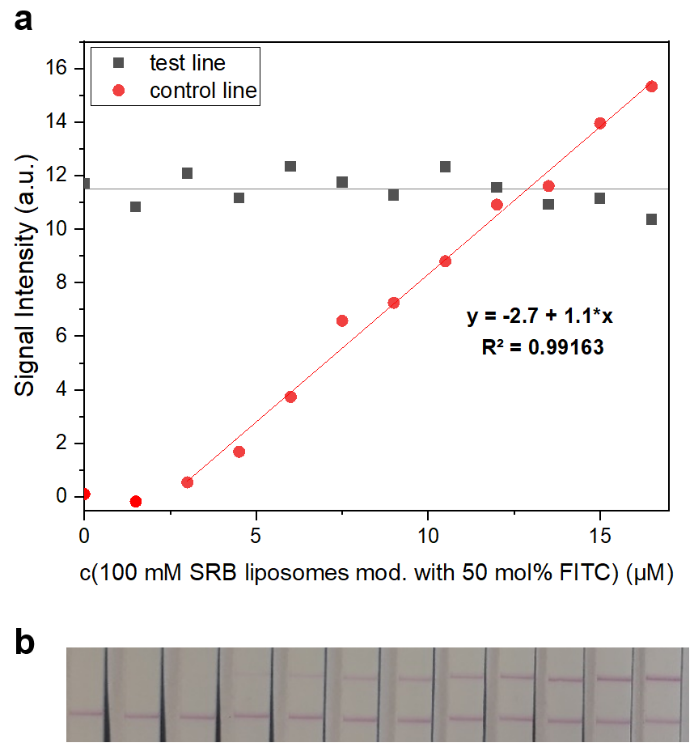


**Fig. S 15** Test strips (b) and their signal intensities (a) of RBD-conjugated liposomes (150 mM SRB, 10 µM total lipid concentration) and control liposomes (100 mM SRB, 50% FITC, 0-16.5 µM total lipid concentration) tested with seronegative pooled human serum (10 v%). Samples were pre-incubated for 15 min at RT and 300 rpm in HSS. ACE2-biotin (3 equivalents per RBD) was added to samples before addition to the test strip. Samples were washed after 5 min (25 µL HSS) and pictures were taken after another 20 min using a Canon EOS 550D camera with a Canon EFS 18-55mm lens. Images were analyzed using ImageJ. n = 1.

Reproducibility of the assay was investigated with seronegative pooled human serum. Six test strips were run under identical conditions, resulting in binding inhibition values between -4% and 4%, with a mean of -2 ± 3% (Fig. S 16).


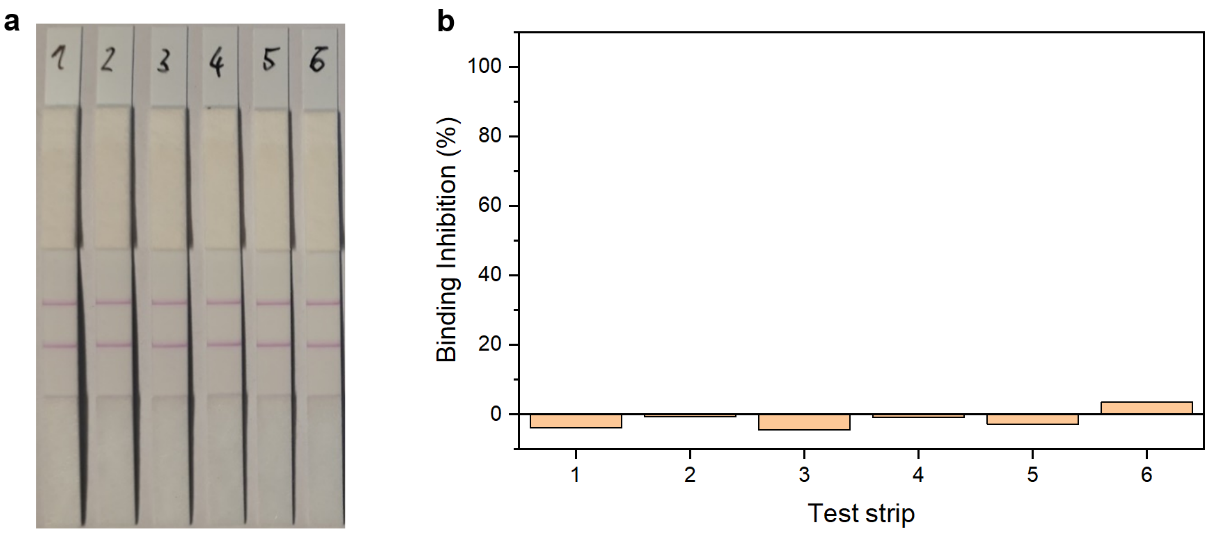


**Fig. S 16** Test strips (a) and binding inhibition values (b) of seronegative pooled human serum samples. n = 1.

Addition of ACE2-biotin before and after pre-incubation of liposomes with serum was investigated for a weakly, intermediate and strongly neutralizing sera. Complete binding inhibition was observed for the strongly neutralizing serum in both cases due to its high neutralization potential (Fig. S 17). The intermediate neutralizing serum showed higher binding inhibition in case of ACE2-biotin addition after pre-incubation, as anticipated. This mimics the realistic interaction of SARS-CoV-2 with the immune system, antibodies present in serum or respiratory fluids being able to neutralize the virus before it reaches potential host cells. If ACE2-biotin is added before pre-incubation instead, it can bind to RBD, and neutralization is dependent on its dissociation. For the weakly neutr. serum a weaker test line was also observed for addition after pre-incubation. However, due to non-specific binding reduced control line intensities were obtained, resulting in false-negatives. Later studies revealed that the non-specific binding is likely caused by formation of agglomerates too large to pass through the pores of the membrane. The use of a membrane with larger pore size (CN95, Sartorius) resulted in reduced non-specific binding compared to the CN150 membrane (Fig. S 20).


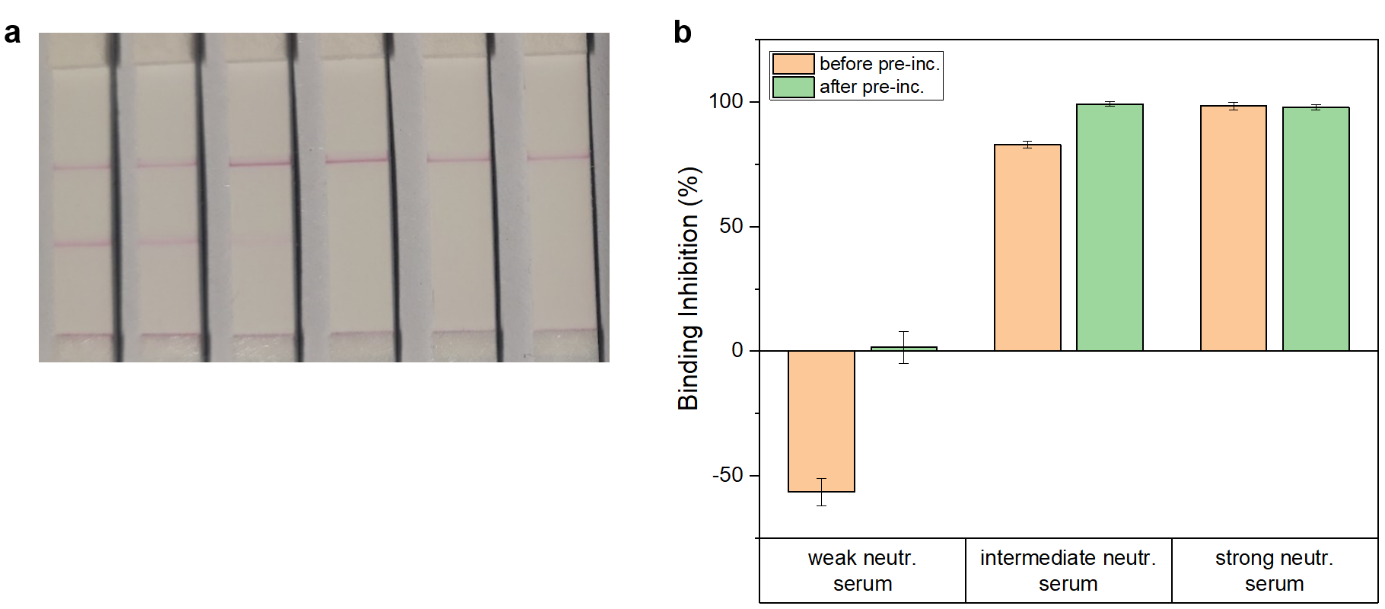


**Fig. S 17** Test strips (a) and binding inhibition values (b) of a weak, intermediate and a strongly neutralizing serum tested with ACE2-biotin addition before or after the pre-incubation of liposomes with serum. n = 1.

Investigation of the influence of the pre-incubation time revealed slightly increased binding inhibition for 15 min over 5 min and 0 min (Fig. S 18). However, 5 min would make for a good compromise in a final product, shortening turn-around time of the assay.


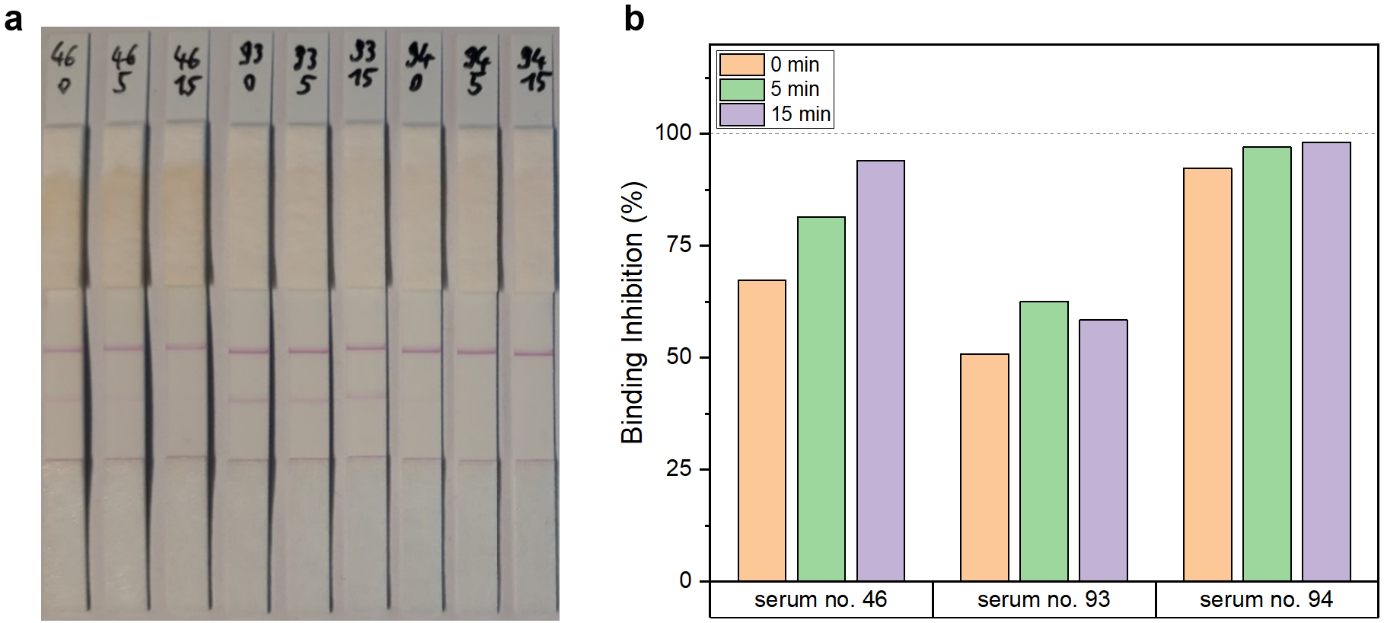


**Fig. S 18** Test strips (a) and binding inhibition values (b) of sera no. 46, 93 and 94 from the serum panel previously tested for binding antibodies against SARS-CoV-2 RBD using the *recom*Line SARS-CoV-2 IgG assay tested in the lateral flow neutralization test with 0 min, 5 min and 15 min pre-incubation of liposomes with serum. n = 1.

24 sera from a serum panel previously tested for binding anti-RBD antibodies with the *recom*Line SARS-CoV-2 assay were analyzed with the established POC neutralization test. Samples included timelines from vaccinated and convalescent donors as well as seronegative samples from patients with other respiratory diseases. The resulting binding inhibition values are displayed in Fig. S 19, details regarding days passed since infection or vaccination can be found in Table S 3.


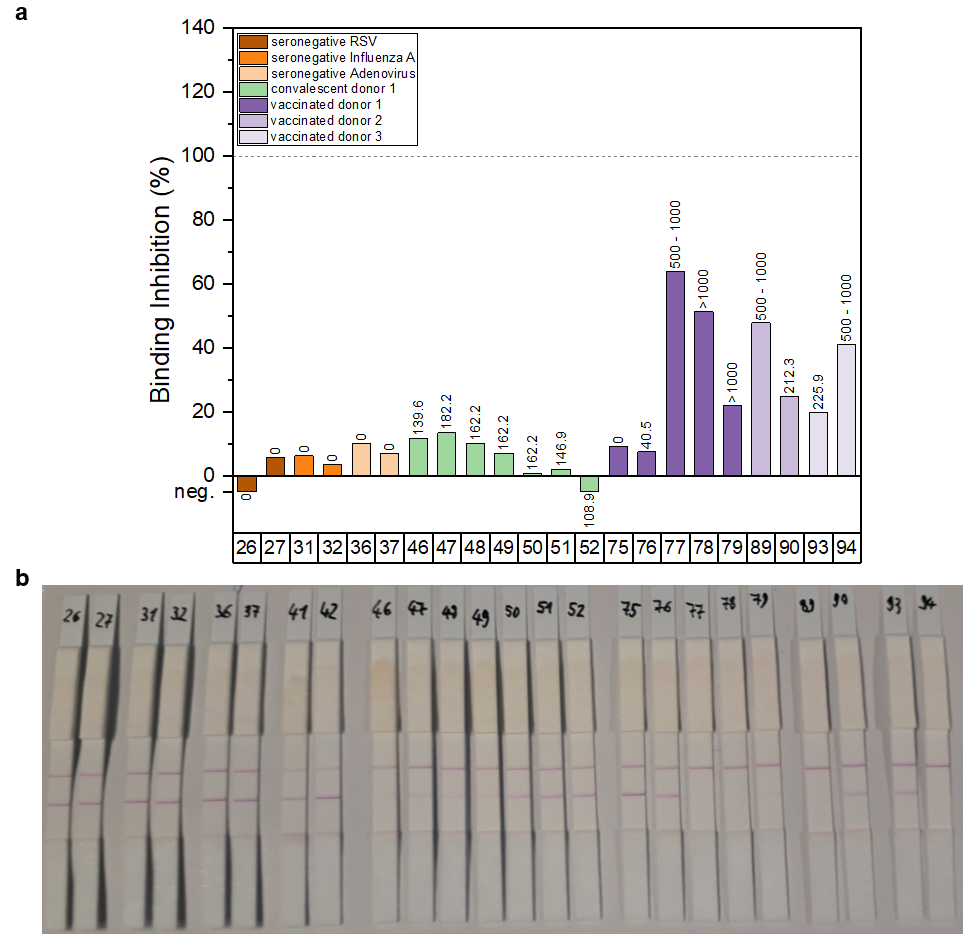


**Fig. S 19** Test strips (b) and binding inhibition values (a) of 24 samples from the Munich Cohort serum panel previously tested for binding antibodies against SARS-CoV-2 RBD using the *recom*Line SARS-CoV-2 IgG assay. n = 1. No binding inhibition values could be determined for sera no. 41 and 42 due to extensive non-specific binding of FITC-conjugated liposomes, resulting in a decreased control line intensity.


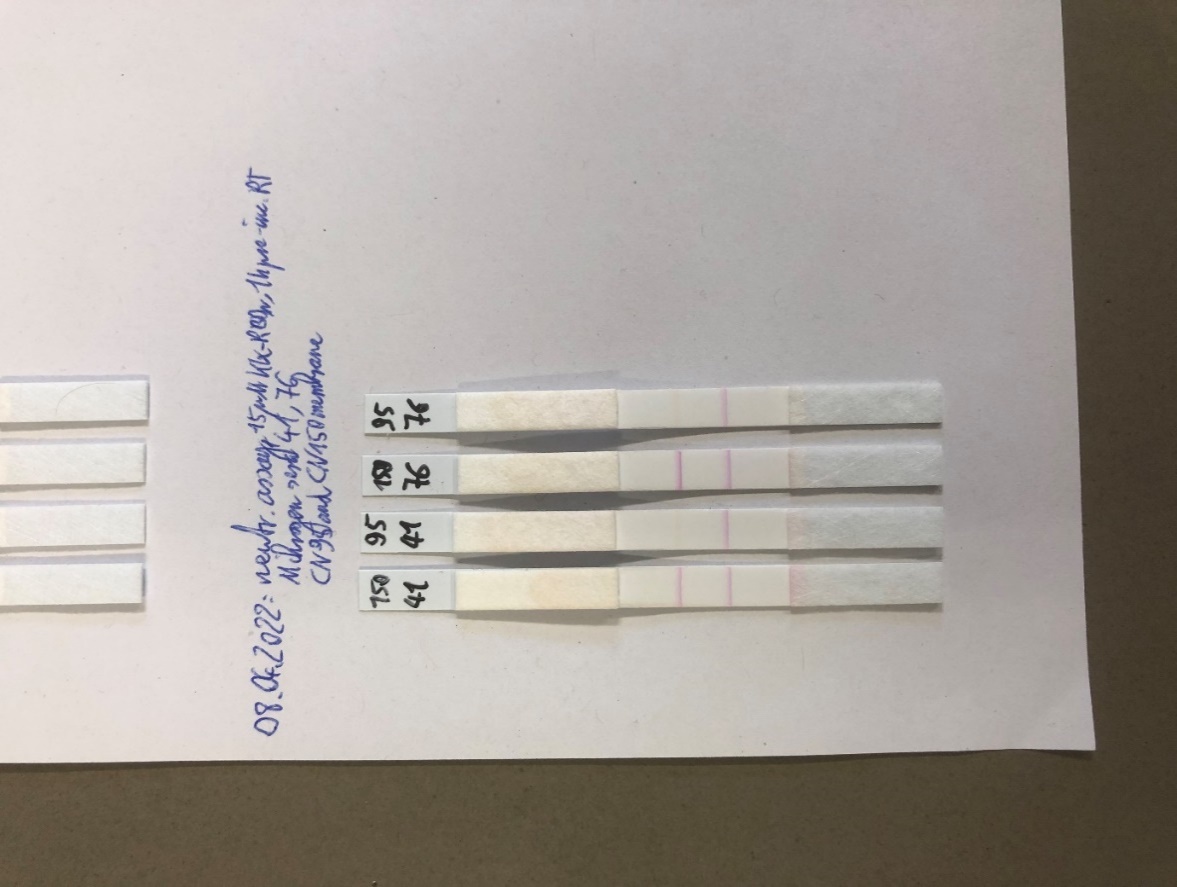


**Fig. S 20** Test strips (1-4 from left to right) run with sera no. 41 (strips 1 and 2) and 76 (strips 3 and 4) from the serum panel previously tested for binding antibodies against SARS-CoV-2 RBD using the *recom*Line SARS-CoV-2 IgG assay. Strips 1 and 3 contained a streptavidin test line and <anti-FITC> control line on CN150 membrane. Strips 2 and 4 contained a streptavidin test line and FITC control line on CN95 membrane.

Six seronegative samples were analyzed to determine the threshold of the assay. The experiment was conducted three times on different days with room temperature varying between 22 and 26 °C. Samples showed binding inhibition values around 0% in most cases, with exception of blood donations 4498 and 4500 with values of 10 ± 7% and -10 ± 3% (Fig. S 21). The average of all 18 measurements (0%) + 3x the standard deviation (11%) was used as preliminary cut-off (33%) for further experiments.

**Fig. S 21** Binding inhibition values of seronegative samples (blood donation 4495-4500) from the serum panel previously tested for binding antibodies against SARS-CoV-2 RBD using the *recom*Line SARS-CoV-2 IgG assay. n = 3.

Investigation of additional 10 seronegative samples suggests good sensitivity of the assay. No binding inhibition was observed for any sample. However, reduced control line intensities for blood donations 4505 and 4507 resulted in exceptionally low values of -24% and -53%.


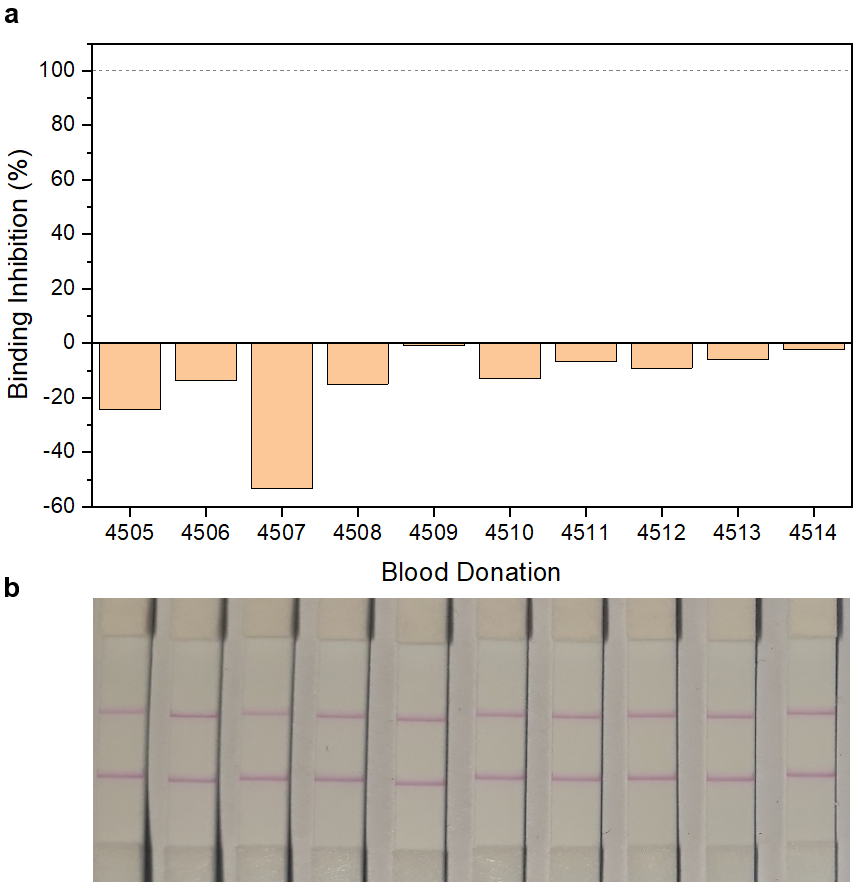


**Fig. S 22** Test strips (b) and binding inhibition values (a) of seronegative samples (blood donation 4505-4514) from the serum panel previously tested for binding antibodies against SARS-CoV-2 RBD using the *recom*Line SARS-CoV-2 IgG assay. n = 1.

**Table S 3** List of used serum samples tested for binding antibodies against SARS-CoV-2 RBD using the *recom*Line SARS-CoV-2 IgG assay. Antibody titers are given as binding antibody units per mL. Vaccinated donor 1 was vaccinated with Spikevax^®^ vaccine from Moderna, vaccinated donors 2 and 3 with Comirnaty^®^ vaccine from BioNTech/Pfizer.

| **No.** | **Sample ID** | **classification** | **comment** | **RBD [BAU/mL]** |
| --- | --- | --- | --- | --- |
| 1 | **blood donation 4495** | seronegative |  | 0 |
| 2 | **blood donation 4496** | seronegative |  | 0 |
| 3 | **blood donation 4497** | seronegative |  | 0 |
| 4 | **blood donation 4498** | seronegative |  | 0 |
| 5 | **blood donation 4499** | seronegative |  | 0 |
| 6 | **blood donation 4500** | seronegative |  | 0 |
| 7 | **blood donation 4501** | seronegative |  | 0 |
| 8 | **blood donation 4502** | seronegative |  | 0 |
| 9 | **blood donation 4503** | seronegative |  | 0 |
| 10 | **blood donation 4504** | seronegative |  | 0 |
| 11 | **blood donation 4505** | seronegative |  | 0 |
| 12 | **blood donation 4506** | seronegative |  | 0 |
| 13 | **blood donation 4507** | seronegative |  | 0 |
| 14 | **blood donation 4508** | seronegative |  | 0 |
| 15 | **blood donation 4509** | seronegative |  | 0 |
| 16 | **blood donation 4510** | seronegative |  | 0 |
| 17 | **blood donation 4511** | seronegative |  | 0 |
| 18 | **blood donation 4512** | seronegative |  | 0 |
| 19 | **blood donation 4513** | seronegative |  | 0 |
| 20 | **blood donation 4514** | seronegative |  | 0 |
| 26 | **RSV 1** | seronegative | RSV infection | 0 |
| 27 | **RSV 2** | seronegative | RSV infection | 0 |
| 28 | **RSV 3** | seronegative | RSV infection | 0 |
| 31 | **Influenza A 1** | seronegative | Influenza A infection | 0 |
| 32 | **Influenza A 2** | seronegative | Influenza A infection | 0 |
| 33 | **Influenza A 3** | seronegative | Influenza A infection | 0 |
| 36 | **Adenovirus 1** | seronegative | Adenovirus infection | 0 |
| 37 | **Adenovirus 2** | seronegative | Adenovirus infection | 0 |
| 38 | **Adenovirus 3** | seronegative | Adenovirus infection | 0 |
| 41 | **Mycoplasm 1** | seronegative | Mycoplasm infection | 0 |
| 42 | **Mycoplasm 2** | seronegative | Mycoplasm infection | 0 |
| 43 | **Mycoplasm 3** | seronegative | Mycoplasm infection | 0 |
| 46 | **convalescent donor 1.1** | convalescent | 27 days after symptom onset | 139.6 |
| 47 | **convalescent donor 1.2** | convalescent | 34 days after symptom onset | 182.2 |
| 48 | **convalescent donor 1.3** | convalescent | 41 days after symptom onset | 162.2 |
| 49 | **convalescent donor 1.4** | convalescent | 50 days after symptom onset | 162.2 |
| 50 | **convalescent donor 1.5** | convalescent | 62 days after symptom onset | 162.2 |
| 51 | **convalescent donor 1.6** | convalescent | 90 days after symptom onset | 146.9 |
| 52 | **convalescent donor 1.7** | convalescent | 132 days after symptom onset | 108.9 |
| 75 | **vaccinated donor 1.1** | vaccinated | before vaccination | 0 |
| 76 | **vaccinated donor 1.2** | vaccinated | 1st vaccination | 40.5 |
| 77 | **vaccinated donor 1.3** | vaccinated | 23 days after 2nd vaccination | 500 - 1000 |
| 78 | **vaccinated donor 1.4** | vaccinated | 86 days after 2nd vaccination | >1000 |
| 79 | **vaccinated donor 1.5** | vaccinated | 174 days after 2nd vaccination | >1000 |
| 89 | **vaccinated donor 2.1** | vaccinated | 60 days after 2nd vaccination | 500 - 1000 |
| 90 | **vaccinated donor 2.2** | vaccinated | 188 days after 2nd vaccination | 212.3 |
| 93 | **vaccinated donor 3.1** | vaccinated | 162 days after 2nd vaccination | 225.9 |
| 94 | **vaccinated donor 3.2** | vaccinated | 3rd vaccination | 500 - 1000 |

The 20 neutralizing sera previously tested in both a pseudovirus neutralization test and the developed HTS neutralization test were subsequently tested in the POC format (Fig. S 23). Obtained binding inhibition values can be found in Table S 2.


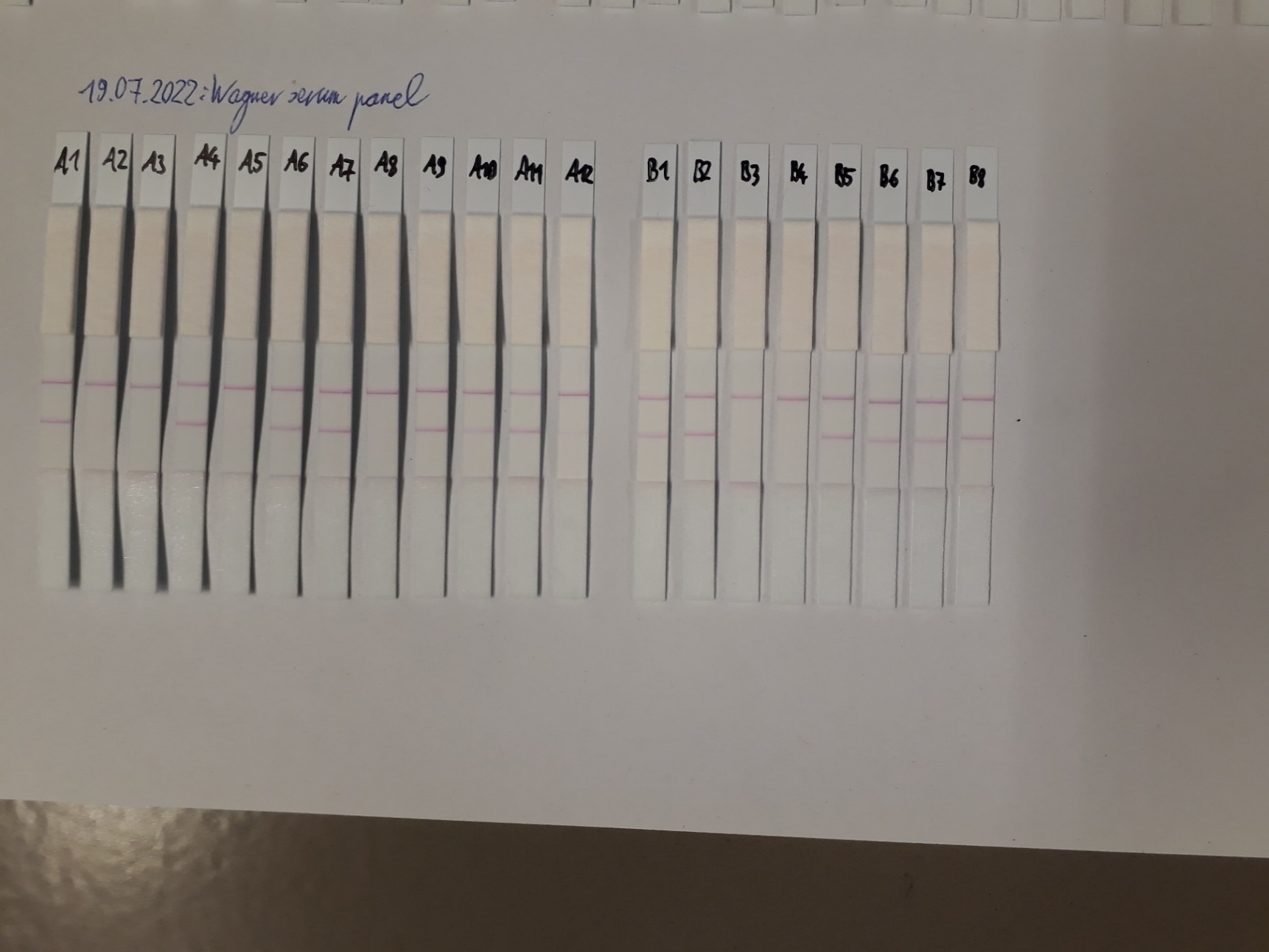


**Fig. S 23** Test strips of 20 neutralizing sera (left to right: nS1-20) tested in the POC neutralization test. n = 1.

## 2.4 Liposome stability

The stability of 150 mM SRB liposomes conjugated to 0.2 mol% RBD was monitored over the course of 10 weeks. Both Z-average and polydispersity index (PDI) showed an increase after 6 weeks coinciding with the observation of precipitation in the storage container (Fig. S 24 a). The liposomes appear to aggregate after >1 month of storage at 4 °C. Leakage was studied by fluorescence measurements of unlysed liposomes in HSS buffer and lysed liposomes in bidest. H_2_O with 30 mM OG. The latter results in the max. fluorescence intensity, which allows for calculation of unlysed fluorescence given as percentage by normalization of the unlysed to the lysed fluorescence. An increase of unlysed fluorescence from 15% to 20% was observed within the first week (Fig. S 24 b), potentially due to partial diffusion of SRB outside of the liposomes, but it may also be within the margin of error. No further increase was observed in the weeks after, suggesting that the liposomes remain stable and do not leak any SRB.


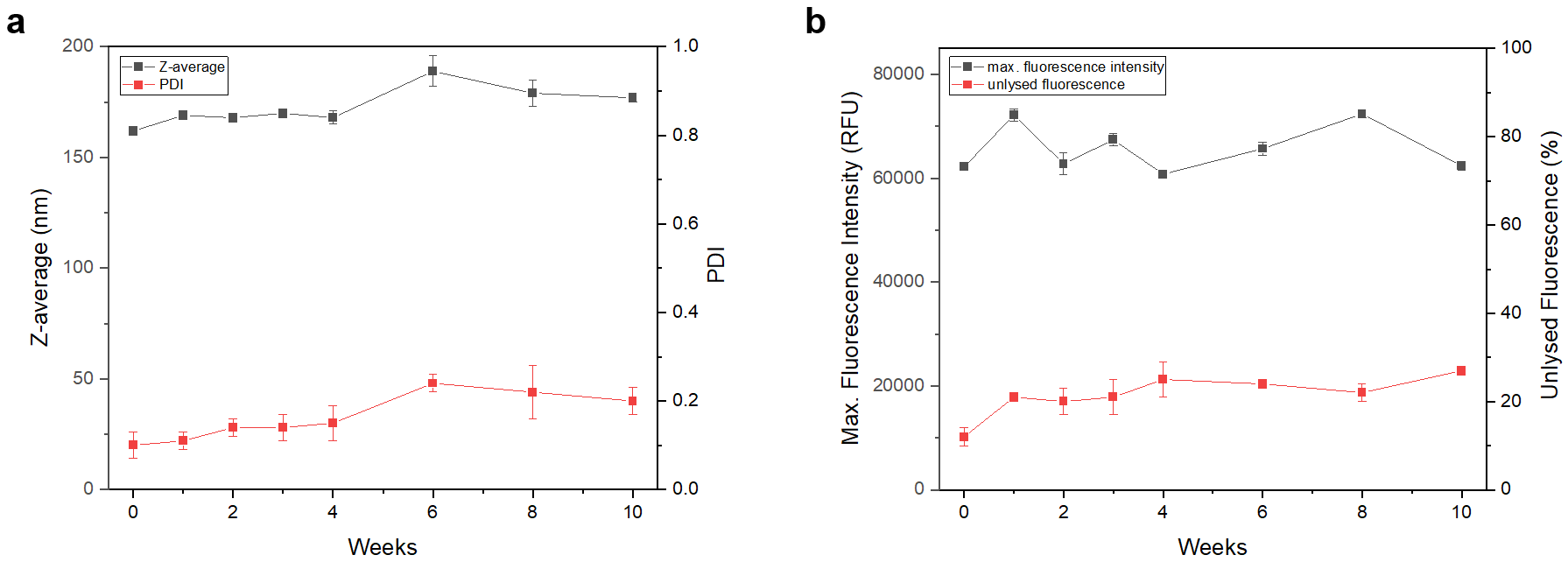


**Fig. S 24** Z-average and PDI (a) and max. fluorescence intensity and unlysed fluorescence (b) of RBD-conjugated liposomes (150 mM SRB) over the course of 10 weeks. Liposomes (900 µM total lipids in HSS) were stored at 4 °C in the dark in between measurements and were vortexed for 20 s before being used. Unlysed fluorescence was calculated by normalization of the fluorescence intensity of liposomes in HSS (5 µM total lipids) to the fluorescence intensity of lysed liposomes (5 µM total lipids, lysed by 10 min incubation with 30 mM OG in bidest. H_2_O).

References

1. Einhauser S, Peterhoff D, Niller HH, Beileke S, Günther F, Steininger P, Burkhardt R, Heid IM, Pfahlberg AB, Überla K, Gefeller O, Wagner R. Spectrum Bias and Individual Strengths of SARS-CoV-2 Serological Tests-A Population-Based Evaluation. Diagnostics. 2021; https://doi.org/10.3390/diagnostics11101843
